# Place cell maps slowly develop via competitive learning and conjunctive coding in the dentate gyrus

**DOI:** 10.1038/s41467-020-18351-6

**Published:** 2020-09-11

**Authors:** Soyoun Kim, Dajung Jung, Sébastien Royer

**Affiliations:** 1 Center for Functional Connectomics, Korea Institute of Science and Technology, Seoul, 02792 Republic of Korea; 2 Center for Neuroscience Imaging Research, Institute for Basic Science (IBS), Suwon, 16419 Republic of Korea; 3 Department of Biological Sciences, Korea Advanced Institute of Science and Technology, Daejeon, 34141 Republic of Korea; 4 Division of Bio-Medical Science and Technology, KIST School, Korea University of Science and Technology, Seoul, 02792 Republic of Korea

**Keywords:** Learning algorithms, Network models, Learning and memory, Hippocampus

## Abstract

Place cells exhibit spatially selective firing fields that collectively map the continuum of positions in environments; how such activity pattern develops with experience is largely unknown. Here, we record putative granule cells (GCs) and mossy cells (MCs) from the dentate gyrus (DG) over 27 days as mice repetitively run through a sequence of objects fixed onto a treadmill belt. We observe a progressive transformation of GC spatial representations, from a sparse encoding of object locations and spatial patterns to increasingly more single, evenly dispersed place fields, while MCs show little transformation and preferentially encode object locations. A competitive learning model of the DG reproduces GC transformations via the progressive integration of landmark-vector cells and spatial inputs and requires MC-mediated feedforward inhibition to evenly distribute GC representations, suggesting that GCs slowly encode conjunctions of objects and spatial information via competitive learning, while MCs help homogenize GC spatial representations.

## Abstract (web summary)

Place cells in the hippocampus fire action potentials at spatially selective firing fields that collectively map the environments. Here, the authors show how these activity patterns develop with experience in mice and determine the importance of competitive learning in this process.

## Introduction

Principal cells in the hippocampus exhibit spatially selective firing fields called “place fields” that collectively map the continuum of positions in environments^1^. How such activity patterns emerge during learning is a fundamental question. The granule cell (GC) population of the dentate gyrus (DG) is the first processing stage of the hippocampal trisynaptic loop; accounts for nearly half of the neurons in the mammalian hippocampus^2^; locally interacts with GABAergic interneurons and mossy cells (MCs), a small population of excitatory neurons located in the hilus (10^4^ MCs versus 10^6^ GCs in rodents)^3–5^; and is largely believed to perform pattern separation^6–10^ on inputs from the entorhinal cortex (EC) via the generation of sparse-orthogonal output patterns^11–15^ and to assist memory formation in the CA3 via powerful GC-to-CA3 synapses^4,5,7,8,16^. Recently, several methods were developed to segregate and assess the spatial representations of specific DG cell types, leading to reports of clear differences between GCs and MCs in terms of the scale, sparseness, stability and remapping of spatial representations^11–15,17^. An emerging picture is that the GCs generate spatial representations that are relatively stable over time and consist predominantly of a small and unique place field, whereas the MCs generate several large place fields that are strongly altered by small changes in the environments; moreover, the GCs efficiently differentiate the environments via the recruitment of small context-specific cell ensembles, whereas the MCs engage large cell ensembles that largely overlap across contexts^11–15,17^. However, the fundamental question on how these diverse spatial representations develop during the learning of an environment remain largely untested.

A theory postulates that the place fields of GCs are generated through competitive learning^18,19^, that is, through the combination of competition between GCs, mediated by feedback inhibition, and Hebbian synaptic plasticity at the level of EC-to-GC inputs. In support of this theory, single place field representations emerge automatically in network models featuring GC competition when EC synaptic weights are recursively updated by Hebbian synaptic plasticity mechanisms^18,19^. Furthermore, object and spatial information is hypothesized to be integrated at the level of the GCs^8,20^. Inputs from both the medial (MEC) and lateral (LEC) divisions of the EC converge onto GCs^5^, and are largely believed to supply diverse types of spatial and non-spatial information. Given that landmark-vector cells (or object-vector cells) in the MEC^21^ and LEC^22^ encode animal spatial relationships with objects, that most cells in the MEC show spatial activity^23^, and that grid cells in MEC exhibit periodic firing fields that convey spatial information related to path integration^24,25^, GCs might be able to bind object and spatial information via the integration of inputs from landmark-vector cells, grid cells and non-grid spatial cells in the EC. Finally, MCs receive direct inputs from the CA3, semilunar GCs and possibly the EC^26,27^ and are particularly well positioned to shape GC activity via both direct and indirect connections. In particular, MC-to-GC feedforward inhibition^27–33^ is expected to affect GC competition and thus competitive learning in the GC network.

To investigate the development of DG spatial representations during the learning of an environment, we recorded putative GCs and MCs over 27 days as mice ran head-fixed on a long treadmill enriched with visual-tactile landmarks^34^. Such an apparatus is particularly suited for differentiating spatial mechanisms^35,36^ and assessing learning effects since the animal trajectory is consistent across trials and the spatial information is reduced to a sequence of individual landmarks fixed on the belt and path integration. We analyzed how spatial representations evolved over days as mice learned the belt layout and, subsequently, how cells encoded other belt layouts. To test the contributions of competitive learning, EC inputs and MCs, we implemented a competitive learning model of the DG that received inputs from EC object-vector cells, grid cells and non-grid spatial cells, and explored the mechanisms and parameters critical to reproduce the experimental data. Our findings suggest that a slow integration of spatial cell and landmark-vector cell inputs, achieved via competitive learning, is the mechanism underlying both the emergence of single place field representations and the continuous mapping of the space by GCs, while an increase in MC feedforward inhibition in the cue locations is required to ensure a uniform distribution of GC representations.

## Results

### Identification of putative GCs and MCs

**Fig. 1:**
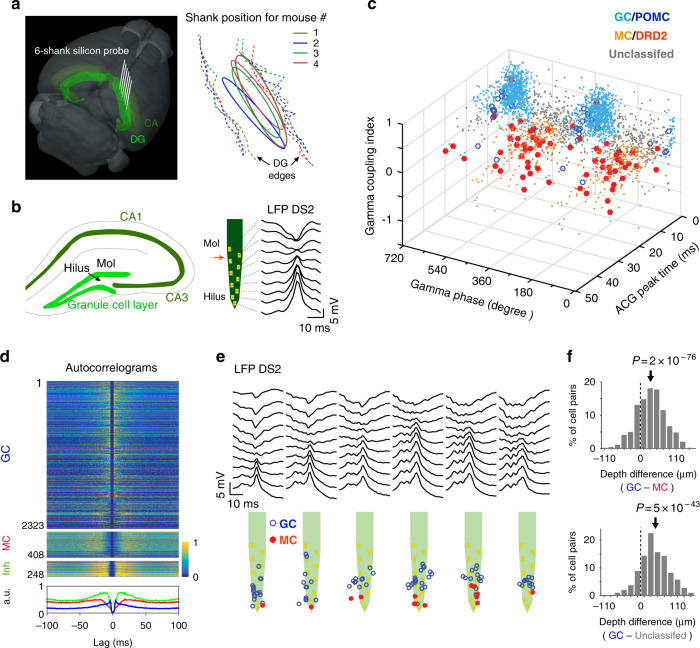
Recording of putative GCs and MCs. **a** Left, 3D representation of the mouse brain (Allen Mouse Brain Institute; www.alleninstitute.org) showing recording electrode configuration in the dentate gyrus (light green). Dark green, cornus ammonis (CA). Right, the electrode positions (ellipsoid) relative to the lateral/medial edges of the granule cell layer (dashed lines) for all mice. **b** Left, scheme showing the location of the hilus and granule cell layer on a coronal section of the hippocampus. Right, layout of recording sites for a shank of the silicon probe and profile of local field potential dentate spike 2 (LFP DS2). Red arrow, position of DS2 reversal. **c** 3D scatter plot for cells’ spike gamma phase, ACG refractory gap and gamma coupling index. Putative MCs (orange dots) and GCs (light blue dots) are identified by overlap with DRD2 (red filled circle) and POMC (blue circle) light-excited cells. **d** Spike ACGs (upper, color-coded representation of individual cell; lower, population average) for putative GCs (blue), MCs (red) and inhibitory cells (green). **e** Layout of LFP DS2 (upper) and putative GCs and MCs (lower) along the silicon probe shanks for one session. **f** Top, distribution of depth differences for all possible pairs of putative GCs and MCs recorded concurrently on the same shanks (*n* = 825 pairs). Bottom, same analysis between GCs and the population of unclassified cells in panel (**c**) (*n* = 301 pairs). Arrows, the means. *P*-values, two-tailed paired *t* test.

We recorded neuronal activity during treadmill running every day for 27 days using a 6-shank silicon probe (64 channels) implanted in the DG of the right brain hemisphere (Fig. 1a, b; Supplementary Fig. 1). A total of 4003 cells were isolated (from 4 mice, 16 sessions per mouse) following standard criteria for unit detection and clustering^37,38^. In addition, to help identify putative GCs and MCs, we used a previous data set in which a subset of GCs and MCs expressed the Chronos opsin (via AAV/hSyn-Flex-Chronos-GFP injections in POMC-Cre and DRD2-Cre mice, respectively) and showed excitatory responses to light stimuli (18 POMC light-excited cells from 3 mice and 33 DRD2 light-excited cells from 2 mice)^17^.

To identify putative GCs and MCs, we assessed differences in cells’ spike autocorrelogram (ACG) and spike relationship with hilar local field potential gamma (30–80 Hz) oscillations (Supplementary Fig. 2a–d), as previously^17^. We measured an ‘ACG refractory gap’ (defined as the duration for the ACG to reach 75% of its peak value; Supplementary Fig. 2a), a gamma coupling index (defined as the difference in gamma power between window periods within [−10 to +10 ms] and outside [+40 to +100 ms] epochs of maximal firing activity; Supplementary Fig. 2d) and the mean spike gamma phase for each cell and examined the cell clustering and overlap with POMC/DRD2 light-excited cells and putative excitatory neurons (detected from short-latency peaks in cell-pair cross-correlograms)^39^. First, some cells were categorized as putative interneurons (*n* = 248) based on their high firing rate, short ACG refractory gap and lack of overlap with putative excitatory neurons (Supplementary Fig. 3a) and were excluded from the next analysis. Then, putative GCs (*n* = 2323) were characterized by short ACG refractory gaps, high gamma coupling indexes, a preference to discharge before the troughs of gamma oscillations and overlap with POMC light-excited cells (Fig. 1c, d; Supplementary Fig. 3b–e). In contrast, putative MCs (*n* = 408) were characterized by large ACG refractory gaps (consistent with in vivo intracellular recording data)^40^, low gamma coupling indexes, a preference to discharge before the peaks of gamma oscillations, and overlap with DRD2 light-excited cells. Consistent with anatomical figures, putative GCs were located closer to the reversal point of LFP type-2 dentate spikes (DS2) above the granule cell layer, while putative MCs were relatively deeper in the positive phase of DS2, i.e., in the hilus^41^ (Fig. 1e); accordingly, on shanks where both putative GCs and MCs were detected, MCs were located on average 31.5 ± 1.5 µm below GCs (*t*_824_ = 20.6, *p* = 2e^−76^, two-tailed paired *t* test, *n* = 47 shanks, average over all GC-MC combinations; Fig. 1f, top). Furthermore, a group of unclassified cells showing short ACG refractory gaps similar to those of GCs and similar gamma relationships to those of MCs (*n* = 168; Supplementary Fig. 3b) were found on average 43.8 ± 2.7 µm below GCs (*t*_300_ = 16.2, *p* = 5e^−43^, two-tailed paired *t* test, *n* = 13 shanks; Fig. 1f, bottom), suggesting these cells might be pyramidal cells from CA3. Finally, a number of cells (*n* = 164) were not included in this classification because the number of spikes discharged was too low (<50 spikes) to reliably measure ACG refractory gaps and gamma phases.

### Progressive transformation of GC firing fields over days

**Fig. 2:**
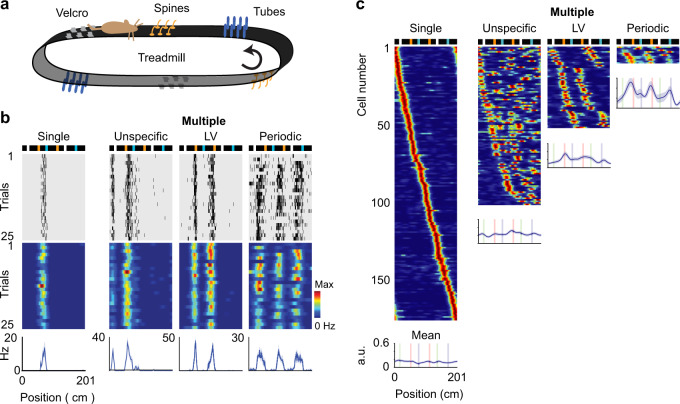
Types of place field activity among GCs. **a** Scheme of the treadmill showing the 3 pairs of landmarks fixed on the belt. **b** Individual cell examples for various types of GC representations: a single-field cell; a landmark-vector (LV) cell (showing two fields matching a type of landmark); a periodic cell (with three periodic fields); and an unspecific cell (with more than one field that are neither LV nor periodic). Top, scheme of the belt; middle, spike raster and color-coded firing rate map; bottom, mean firing rate. **c** Color-coded, firing rate maps of GCs from all sessions, sorted according to field positions and grouped by type of representation. Line plots, average of firing rate maps for each type of representation (lines, the mean; shadows, s.e.m; *n* = 178 single, 105 unspecific, 53 LV and 11 periodic cells). The color scale is the same as that used in (**b**).

To investigate place cell activity during familiarization with the belt layout, mice were trained to run head-fixed for a water reward on an empty 150-cm-long belt for a week and then were introduced to a 201-cm-long belt displaying visual-tactile landmarks (Fig. 2a; Supplementary Fig. 4). The landmarks consisted of 5-cm-long arrays of small erect objects that lined both edges of the belt and provided visual-tactile stimulation to both sides of the mice. We used three types of landmarks: an array of shrink tubes, an array of Velcro pieces, and an array of glue spines. To detect cell activity associated with a given landmark, each landmark was fixed to two locations of the belt. A water reward was delivered through a lick port on every trial (belt cycle) at the same belt position (position 0 cm).

We distinguished four types of place field activity among GCs (Fig. 2b, c): (1) single place field cells; (2) unspecific cells, which exhibited several firing fields with no apparent periodicity or relationship with landmarks; (3) landmark-vector (LV) cells, which, similar to LV cells in the EC and CA1 (refs. ^21,35^), encoded spatial relationships with landmarks; and (4) periodic cells, which exhibited three periodic firing fields with similar periodicity but various offsets.

**Fig. 3:**
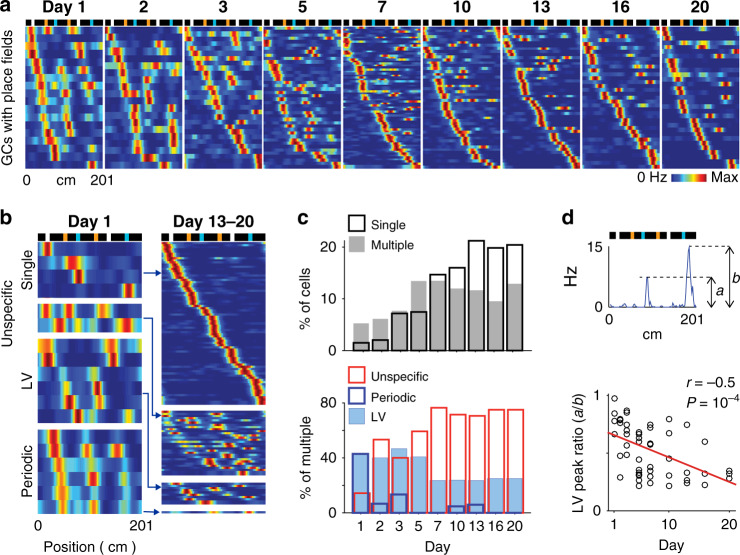
Progressive transformation of GC firing fields across days. **a** Color-coded, firing rate maps of GCs across days. Only the GCs showing firing fields are displayed for clarity. The rows of the matrices correspond to individual GCs and are sorted according to firing field positions. Top, scheme of the belt. **b** Color-coded, firing rate maps of GCs on day 1 (left) and on days 13, 16, and 20 combined (right), for each type of GC firing field. The color scale is the same as that used in (**a**). **c** Upper, proportion of GCs with a single field (black) and multiple fields (gray) across days. Lower, proportion of LV (light blue), periodic (dark blue) and unspecific GCs (red), among multiple-field GCs, across days. **d** Upper, definition of LV peak ratio as the ratio between LV fields’ peak firing rates (smaller peak over larger peak). Lower, distribution of LV peak ratios across days. Each dot is the LV peak ratio of one LV cell. Red line, linear fit. *n* = 53, *r* = −0.5, *p* = 1e^−4^, Pearson’s correlation, two-tailed *t* test.

GC representations gradually transformed across days (Fig. 3a–c). On day 1, a few GCs exhibited place fields (1.6 ± 0.5% single and 5.8 ± 2.2% multiple-field cells (*n* = 14 GCs)) and were mostly LV cells and periodic cells (Fig. 3b, c; among multiple-field cells, 43.3 ± 19.0% LV, 44.4 ± 22.2% periodic and 12.2 ± 6.2% unspecific cells). Then, the fraction of GCs with place fields progressively increased via an increase in unspecific and single-field GCs, reaching a plateau after 5 and 10 days, respectively, while the fraction of LV cells and periodic cells decreased (Fig. 3b, c; for days 13–20, 20.2 ± 3.3% single and 11.8 ± 1.6 multiple-field cells (*n* = 41 GCs); among multiple-field cells, 21.0 ± 5.3% LV, 1.7 ± 1.7% periodic and 77.4 ± 5.6% unspecific cells). Moreover, the peak firing rates of the two fields of LV cells became increasingly uneven (Fig. 3d; *n* = 53, *r* = −0.5, *p* = 1e^−4^, Pearson’s correlation; peak rate ratio on day 1 versus days 13–20, 81.8 ± 5.8% (*n* = 6 LV cells) versus 36.8 ± 5.3% (*n* = 10 LV cells), *t*_14_ = 5.5, *p* = 8e^−5^, two-tailed unpaired *t* test).

### Emergence and extinction of firing fields within sessions

**Fig. 4:**
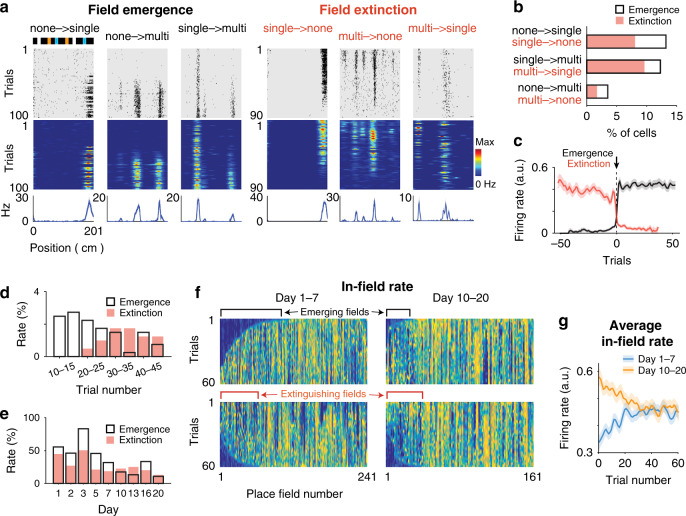
Emergence and extinction of firing fields within sessions. **a** Individual cell examples for field emergences and field extinctions within a session for GCs converting between no field, single-field and multiple-field conditions. Top, scheme of the belt; middle, spike raster plot and color-coded firing rate map; bottom, mean firing rate. **b** Proportion of GC conversion types for field emergences (black, *n* = 41 cells) and field extinctions (salmon, *n* = 21 cells). Proportions are relative to the number of GCs with place fields. **c** Average in-field firing rate (lines, the mean; shadow, s.e.m) for field emergences (black, *n* = 125 cells) and field extinctions (salmon, *n* = 88 cells), after aligning to emergence (or extinction) trials of in-field firing rate vectors. Note that field emergence is relatively instantaneous, as the in-field firing rate reaches an immediate plateau, while field extinction is preceded by a gradual decrease in the in-field firing rate. **d** Proportion of field emergence (black, *n* = 53 cells) and field extinction (salmon, *n* = 30 cells) events as a function of session trials. **e** Proportion of field emergence (black, *n* = 125 cells) and field extinction (salmon, *n* = 88 cells) events as a function of days. **f** Color-coded representation of in-field firing rate vectors for all place fields on days 1–7 (left) and days 10–20 (right). In-field firing rate vectors are sorted to emphasize field emergences (upper, sorted by trial number for which cumulative sums >20% of vector integrals) and field extinctions (lower, using the same sorting method in the reversed direction). The color scale is the same as that used in (**a**). **g** Average in-field firing rate (lines, the mean; shadow, s.e.m) over trials for days 1–7 (blue, *n* = 241 place fields) and days 10–20 (yellow, *n* = 161 place fields).

The increase of single-field representations and the reduced proportion of LV and periodic cells implies that new place fields emerged and that existing place fields became extinct. Both place field emergence and extinction events could be observed within sessions (see Methods, Fig. 4a) and produced preferentially incremental changes in the number of firing fields in each cell (Fig. 4b, *F*_1,48_ = 4.5, *p* = 0.04, two-way ANOVA). Place field emergences were characterized by a steep rise of the in-field rate to a plateau value (Fig. 4c) and occurred preferentially at the beginning of the sessions (Fig. 4d; percent of events before versus after trial 30, 75.6 ± 10.2% versus 24.4 ± 10.2%, *t*_15_ = 2.5, *p* = 0.02, two-tailed paired *t* test), while place field extinctions were preceded by gradual decreases in the in-field firing rate (Fig. 4c; change in firing rate from trial −50 to −1 before extinction, *r* = −0.75, *p* = 5e^−10^, Pearson’s correlation) and mostly occurred late in the sessions (Fig. 4d; percent of events before versus after trial 30, 20.8 ± 8.9% versus 79.2 ± 8.9%, *t*_15_ = −3.3, *p* = 5e^−3^, two-tailed paired *t* test).

Importantly, emergence and extinction rates changed across days, which was consistent with the gradual transformation of GC representations. While both emergence and extinction rates decreased across days (Fig. 4e; *F*_8,42_ = 3.7, *p* = 2e^−3^, two-way ANOVA, emergence rate across days 1–7 versus days 10–20, 43.1 ± 6.6% versus 19.9 ± 5.1%, *t*_24_ = 2.8, *p* = 0.01, two-tailed unpaired *t* test; extinction rate, 34.6 ± 5.6% versus 21.5 ± 3.5%, *t*_24_ = 2.0, *p* = 0.06, two-tailed unpaired *t* test), the emergence rate was initially higher than the extinction rate and reached an equivalent level after 7 days (emergence versus extinction, days 1–7, *t*_4_ = 5.0, *p* = 7e^−3^; days 10–20, *t*_3_ = −0.26, *p* = 0.81, two-tailed paired *t* test), matching the increase in and stabilization of place cells observed in Fig. 3c. This effect was also observable in the matrix concatenation of in-field firing rates for all GC place fields, sorted by time of field emergence or extinction (Fig. 4f) and was also revealed by distinct profiles of average in-field rate for days 1–7 and 10–20 (Fig. 4g).

### Changing the belt

**Fig. 5:**
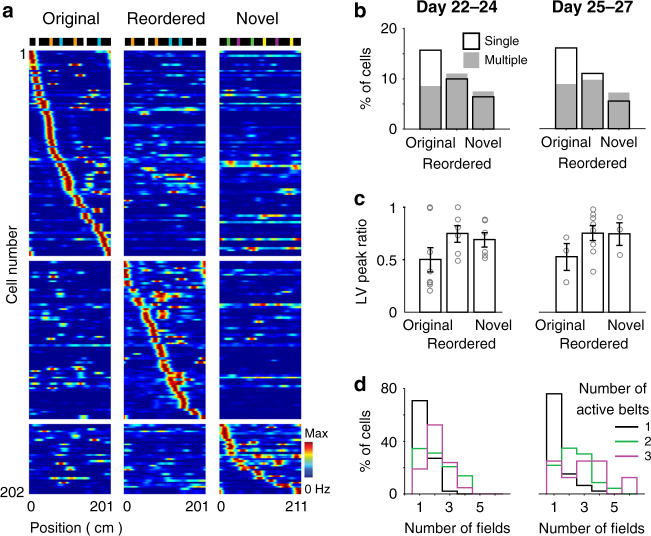
GC encoding of other belt layouts. **a** Firing rate maps of GCs for the original (left), reordered (center) and novel (right) belts. Top, scheme of the belt layouts. Color-coded, each row represents the firing rate maps of a GC for the 3 belts, normalized by the peak firing rate of the cell across all belts. Only GCs exhibiting place fields in at least one of the belts are displayed. GCs are sorted according to the type of belt and the belt position of the largest firing fields. **b** Fraction of GCs with single (black) and multiple (gray) place fields for each belt, during early (days 22–24, left; *n* = 67 single and 50 multiple-field cells) and late (days 25–27, right; *n* = 62 single and 41 multiple-field cells) periods on the new belt layouts. **c** LV peak ratio (circle, individual cell; bar, the mean; error bar, s.e.m.) of LV cells for the 3 belts, during early (left, *n* = 18 LV cells) and late (right, *n* = 13 LV cells) periods on the new belt layouts. Note that the magnitude of the two firing fields is more similar in the reordered and novel belts than in the original belt. **d** For the groups of GCs that are active in 1 (black), 2 (green), and 3 (purple) belts, the distribution of the number of fields per cell (in the belt with the largest number of fields), during early (left) and late (right) periods on the new belt layouts. Note that cells exhibiting multiple place fields tend to represent several belts, while cells exhibiting a single place field tend to represent only one belt, *t*_96_ = 4.7, *p* = 9e^−6^ (days 22–24), *t*_83_ = 4.8, *p* = 6e^−6^ (days 25–27), two-tailed unpaired *t* test).

The gradual transformation of GC representations might be associated with the development of an engram specific to the particular features of the belt. To test the dependence of place cell activity on belt features, after day 21, we recorded the same neurons across three consecutive sessions using three distinct belt layouts: the original belt layout; a reordered belt, presenting the same landmarks as the original belt but in a rearranged order; and a novel belt, which was a different length (211 cm) and presented a new set of landmarks (Fig. 5a).

Consistent with the idea that an engram specific to the layout of the original belt was created, GCs exhibiting single place fields were less frequent in sessions using the reordered and novel belts than in sessions using the original belt (Fig. 5b; original belt, 15.4 ± 2.3%, 15.8 ± 1.6%; reordered belt, 10.6 ± 2.3%, 12.7 ± 3.3%; novel belt, 8.1 ± 2.5%, 6.7 ± 2.2% for days 22–24 and 25–27 respectively; *F*_2,68_ = 6.0, *p* = 4e^−3^, two-way ANOVA; original vs. reordered, *t*_23_ = 1.7, *p* = 0.10; original vs. novel, *t*_23_ = 5.0, *p* = 4e^−5^, ad hoc two-tailed paired *t* test; across days 22–27); for LV cells, the magnitude of the two firing fields was more similar in sessions using the reordered and novel belts than in sessions using the original belt (Fig. 5c; peak rate ratio, original belt, 0.50 ± 0.11 (*n* = 8, days 22–24), 0.53 ± 0.13 (*n* = 3, days 25–27); reordered belt, 0.74 ± 0.08 (*n* = 6, days 22–24), 0.75 ± 0.07 (*n* = 8, days 25–27); novel belt, 0.69 ± 0.07 (*n* = 6, days 22–24), 0.74 ± 0.11 (*n* = 3, days 25–27); *F*_2,30_ = 3.5, *p* = 0.04, two-way ANOVA; original vs. reordered, *t*_23_ = 2.5, *p* = 0.02, original vs. novel, *t*_18_ = 1.8, *p* = 0.08, ad hoc two-tailed unpaired *t* test; average across days 22–27). However, the fraction of single-field cells was higher in sessions using the reordered belt than in those using the new belt (*t*_23_ = 2.1, *p* = 0.04, ad hoc two-tailed paired *t* test), suggesting that the engram may have helped place field generation for other belts according to the degree of belt similarity.

Furthermore, a relationship was apparent between the number of place fields and the number of belts represented by each cell (Fig. 5d). Cells exhibiting multiple place fields tended to show place fields for several belts, while cells exhibiting single place fields tended to be active in only one belt (average number of belts represented by multiple-field cells, 2.06 ± 0.11 (*n* = 50, days 22–24), 2.02 ± 0.12 (*n* = 41, days 25–27), and by single-field cells, 1.38 ± 0.09 (*n* = 48, day 22–24), 1.30 ± 0.10 (*n* = 44, day 25–27); *t*_96_ = 4.7, *p* = 9e^−6^, *t*_83_ = 4.8, *p* = 6e^−6^, for days 22–24 and 25–27 respectively, two-tailed unpaired *t* test).

### Representations of MCs and CA3 cells

**Fig. 6:**
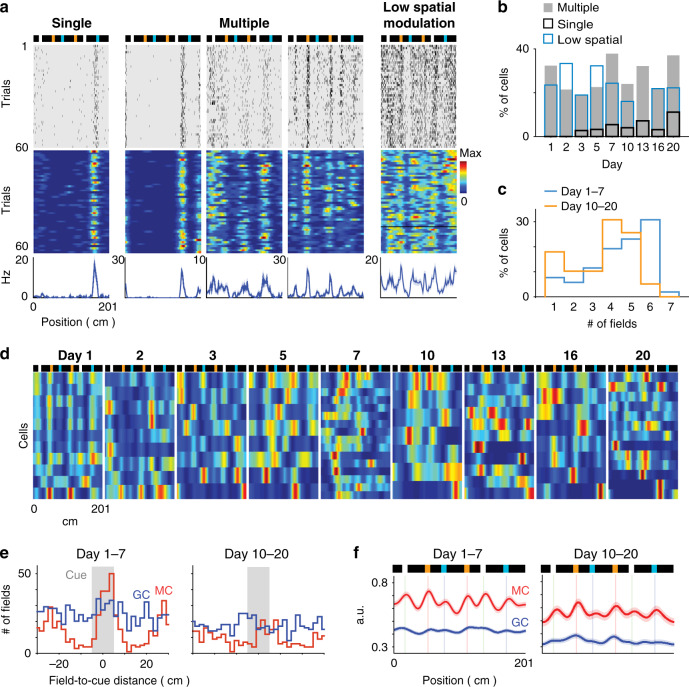
MC overrepresentation of landmarks. **a** Individual cell examples for various types of MC representations. From left to right, a single-field cell, three multiple-field cells, and a cell with low spatial modulation. Top, scheme of the belt; middle, spike raster plot and color-coded firing rate map; bottom, the mean firing rate. **b** Proportion of MCs with single fields (black), multiple fields (gray) and low spatial modulation (blue), across days. **c** Distribution of the number of fields per cell for days 1–7 and days 10–20 among MCs exhibiting place fields. The number of place fields per cell was reduced across days (*t*_89_ = 2.8, *p* = 7e^−3^, two-tailed unpaired *t* test). **d** Color-coded rate maps for MCs exhibiting firing fields across days. The rows of the matrices correspond to individual MCs and are sorted according to firing field positions. The color scale is the same as that used in (**a**). **e** Distribution of field-to-landmark distances for GC (blue) and MC (red) place fields for days 1–7 (left) and days 10–20 (right). **f** Average of GC (blue) and MC (red) rate maps (line, the mean; shadow, s.e.m.) for days 1–7 (left, *n* = 1159 GCs and 181 MCs) and days 10–20 (right, *n* = 540 GCs and 112 MCs). Note the increase in MC (but not GC) activity in landmark positions.

We distinguished three types of firing activity patterns among MCs: (1) cells with a single place field, (2) cells with multiple place fields, and (3) cells with relatively high firing rates (i.e., a mean firing rate >3 Hz) but low spatial modulation (Fig. 6a, see Methods). In contrast to GCs, the fraction of MCs showing firing activity on the belt was initially high (70.6 ± 10.7% of MCs compared to 7.3 ± 2.7% of GCs on day 1; *t*_3_ = 6.4, *p* = 7e^−3^, two-tailed paired *t* test) and did not change significantly across days (Fig. 6b; days 1–3 versus days 13–20, 67.3 ± 4.3% (*n* = 12) versus 64.5 ± 7.5% (*n* = 12); *t*_22_ = 0.3, *p* = 0.8, two-tailed unpaired *t* test); and for all sessions, spatially modulated MCs showed mostly multiple firing fields (Fig. 6b–d), even though the number of fields per cell decreased across days (Fig. 6c; days 1–7 versus days 10–20, 4.44 ± 0.22 versus 3.51 ± 0.25 fields per cell; *n* = 52 and 39, respectively, *t*_89_ = 2.8, *p* = 7e^−3^, two-tailed unpaired *t* test).

Unexpectedly, MC firing fields were strongly modulated by the landmarks, as they were repeated at multiple landmark positions with little spatial offset from the landmarks (Fig. 6d). Accordingly, both the distribution of field-to-landmark distances and the averaged cell firing rate maps showed clear peaks in landmark positions for MCs, but not for GCs, an effect that was reduced but still prominent during later sessions (Fig. 6e, f; fraction of fields with peak <5 cm from landmarks, days 1–7, MCs, 70.2 ± 5.0% (*n* = 19), GCs, 50.0 ± 5.4% (*n* = 19), *t*_36_ = 2.8, *p* = 9e^−3^; days 10–20, MCs, 49.6 ± 5.8% (*n* = 12), GCs, 43.4 ± 4.3% (*n* = 16), *t*_26_ = 0.9, *p* = 0.39; days 1–7 versus days 10–20, MCs, *t*_29_ = 2.6, *p* = 0.01, GCs, *t*_33_ = 0.9, *p* = 0.40, two-tailed unpaired *t* test). Given such difference between MC and GC spatial activities, MC spatial activity was likely not predominantly generated by GC inputs (consistent with ref. ^14^).

Firing fields in the small subset of putative CA3 cells resembled the ones of the GC population (*n* = 34 single fields, *n* = 43 multiple unspecific fields, and *n* = 7 LV fields) except that we could not detect cells with periodic fields (Supplementary Fig. 5a). The proportion of cells exhibiting firing fields and the ratio of multiple versus single fields were higher than for the GC population (cells with place fields, CA3, 58.1 ± 5.6%, GCs, 31.9 ± 4.9%, *t*_4_ = 4.9, *p* = 0.01; multiple over single ratio, CA3, 1.68 ± 0.34, GCs, 0.55 ± 0.04, *t*_4_ = 3.3, *p* = 0.03, two-tailed unpaired *t* test, sessions from days 13–20), consistent with previous reports^13–15^, and unlike the GCs, the spatial representation did not increase drastically across days (151.5 ± 14.4% increase between days 1–3 and days 13–20, versus 370.1 ± 82.4% for GCs, *t*_6_ = 2.6, *p* = 0.06, two-tailed unpaired *t* test), and the LV firing fields almost vanished after a few days, consistent with the relative paucity of CA3 LV cells in familiar environments^35^ (Supplementary Fig. 5b, c). The average firing rate map showed only slight increases near landmark locations in early sessions (Supplementary Fig. 5d), suggesting that MC modulation by the landmarks was likely not caused by CA3 input but by other inputs such as semilunar granule cells^26^ or the EC^27,29^.

### Modeling the increase in GC single place fields

**Fig. 7:**
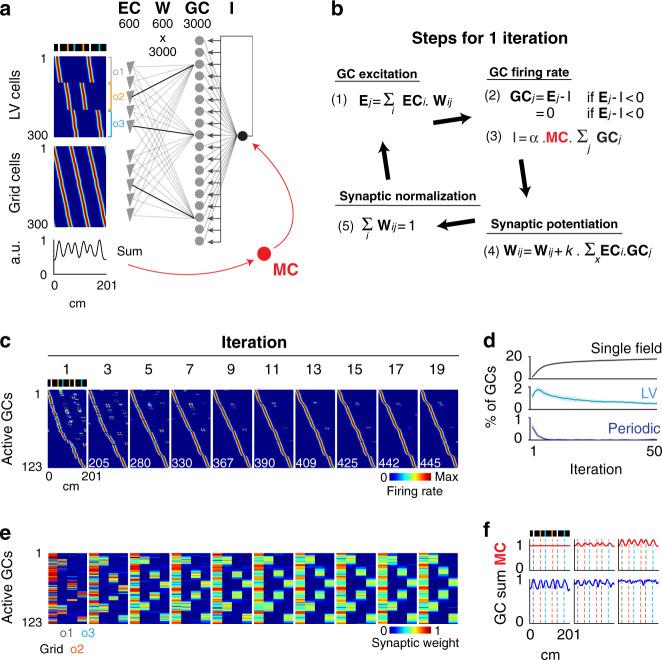
Modeling of single-field emergence **via** competitive learning. **a** Model architecture. A total of 3000 GCs receive excitatory inputs from 300 LV cells and 300 grid cells from the EC and are subjected to feedback inhibition. The EC-to-GC synaptic weight matrix is referred to as Wij. The threshold used as feedback inhibition is referred to as I. Color coded, rate maps of EC LV cells (upper) and grid cells (lower). The firing fields were generated with Gaussian functions. LV cells encode various distances to landmarks, while grid cells have various spatial phases and the same periodicity as periodic cells. In red, the effect of MC feedforward inhibition is added to the model. The spatial modulation of MC activity is assumed to be proportional to the dynamic range of EC average activity. The color scale is the same as that used in (**c**). **b** All operations executed during one model iteration. (1) The excitation Ej received by the GCj in a given position. (2) The firing rate of GCj and (3) the feedback inhibition I in that position. The value of I is estimated numerically by finding the value among a range of I values that best satisfies relations (2) and (3). (4) The potentiation of synaptic weights, proportional to the level of cells’ co-firing throughout the belt. (5) The normalization of synaptic weights. **c** Transformation of spatial representations across iterations. Color-coded representation of rate maps for active GCs, across iterations. Active cells have a mean activity >0 and are sorted according to firing field positions. Note the increase in the number of active GCs (lower left numbers) and the transformation of representations from multiple to single place fields. **d** Evolution of the fraction of single, LV and periodic fields across iterations. **e** For each active GC, the color-coded representation of the total synaptic weight contributed by grid cells and by each population of LV cells (encoding a specific landmark) is shown across iterations using the same GC ordering as in (**c**). Note that synaptic inputs initially originate from one source and are progressively redistributed equally among grid cells and LV cells. **f** Effect of the distinct magnitude of MC modulation on the GC mean activity.

The DG has been modeled as a competitive network in which discrete place field representations are generated via competitive learning^18,19^. To test whether competitive learning could produce the gradual increase in single-field GCs and the decrease in LV and periodic GCs, we first implemented a model of DG in which 3000 GCs received excitatory inputs from 300 EC grid cells (with spatial periodicity similar to that of the periodic cells) and 300 EC LV cells and, in addition, were subjected to feedback inhibition (Fig. 7a (gray and black); Supplementary Fig. 6a). For a given belt position, the excitation (*E*) received by a GC was the weighted sum of EC cell activity, the feedback inhibition (*I*) was proportional to the sum of GC activity, and the firing rate of a GC was equal to *E*–*I* (if *E* > *I*) or 0 (if *E* < *I*). EC-to-GC synaptic weights were initially allocated randomly according to a gamma distribution and then modified on each iteration by two operations: Hebbian synaptic potentiation, proportional to the degree of GC–EC coactivation, and normalization of the total synaptic weight for each GC^42–44^ (Fig. 7b (black); Supplementary Fig. 6b).

We found that two parameters critical to reproduce the gradual increase in single-field GCs were the initial repartition of EC-to-GC synaptic weights and the strength of the feedback inhibition. For a specific range of feedback inhibition strength and sparsity of initial inputs, the model could reproduce the initial preponderance of periodic and LV GCs and the asymptotic increase in single-field GCs (Fig. 7c, d; Supplementary Fig. 6c, d). When the sparsity of initial inputs was set too low (i.e., each GC receives substantial excitation from several EC cells), an asymptotic decrease in single-field GCs was observed over time, whereas when it was set too high (i.e., each GC is mostly excited by one EC neuron), the fraction of GCs that developed single place fields was too high. When the feedback inhibition was set too low, the fraction of GCs with place fields (single, periodic or LV) was too high.

Importantly, the transformation of GC representations was accompanied by a specific reconfiguration of EC-to-GC synaptic weights. First, the overall change in synaptic weights (estimated as the vector Euclidean distance $[ {\sum ( {{{W}}_{{{ij}}}( {{{i}} + {{1}}} )-{{W}}_{{{ij}}}( {{i}} )} )^{{2}}} ]^{1/2}$ between consecutive iterations) exhibited an exponential decrease, suggesting that the plateauing of single-field GCs corresponded to a stabilization of the synaptic weights (Supplementary Fig. 6e, black). Importantly, the overall synaptic change measured just after the synaptic potentiation operation did not show such a drastic decrease (Supplementary Fig. 6e, red), indicating that the synaptic weights were stabilized because the effects of synaptic potentiation were progressively matched and reversed by the effects of synaptic normalization. Other parameters such as the inhibition threshold and *E*/*I* ratio (excitation divided by inhibition) did not show trends that could explain the asymptotic increase in single-field GCs (Supplementary Fig. 6f, g). Second, while most excitatory input to a GC was initially contributed by one EC neuron as a result of the initial sparse input setting, excitatory input to a GC was progressively shared by multiple EC neurons, and each GC progressively received, in comparable amount, inputs from grid cells and LV cells (Fig. 7e). Hence, this simple competitive learning model could reproduce the transformation of GC representations, from periodic and LV place fields to single place fields, through the progressive integration of grid cell and LV cell inputs.

### Modeling the contribution of MCs to GC representations

Given that MC average activity was increased in landmark locations and that an important contribution of MCs is believed to be feedforward inhibition^27–32^ (but see ref. ^33^), we tested how an increase of inhibition in landmark locations affects GC representations. Since MCs presumably receive EC information both directly and indirectly^27^, we assumed that average MC activity reflects average EC activity and modulates the threshold parameter *I* of the model (Fig. 7a, b (red); Supplementary Fig. 7).

In contrast to the experimental findings, average GC activity progressively increased in landmark positions when the inhibition was not modulated by MCs (Fig. 7f; Supplementary Fig. 7c). However, this effect decreased as MC modulation of inhibition was strengthened, such that GCs could evenly represent all belt locations (Fig. 7f; Supplementary Fig. 7d–f). Hence, increasing inhibition in locations associated with larger excitation is necessary to achieve uniform mapping of the space via competitive learning, and a role of MC feedforward inhibition might be to enable the uniform mapping of the space by GCs.

### Modeling the increase in multiple unspecific GC place fields

**Fig. 8:**
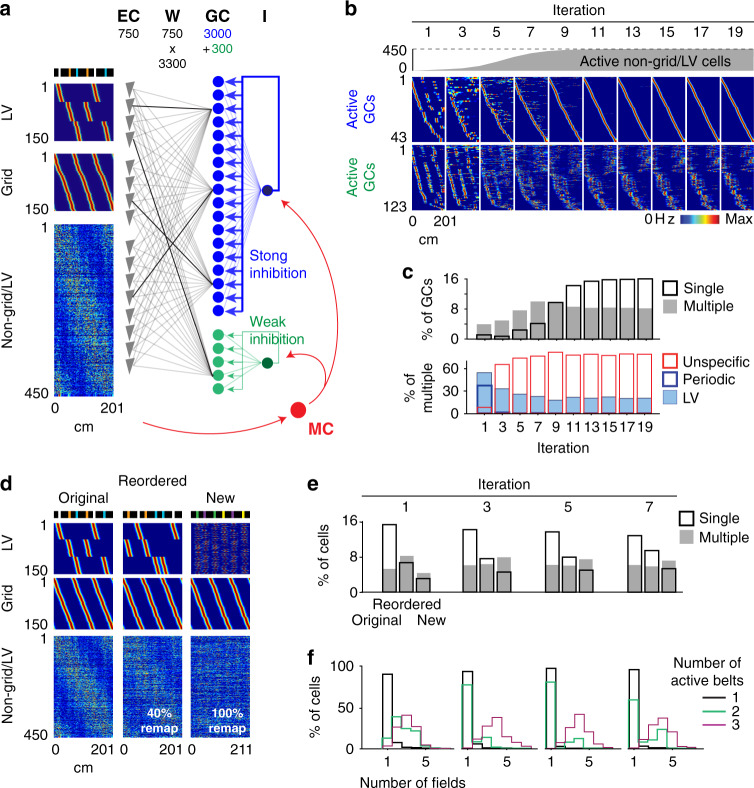
Modeling multiple unspecific fields and remapping experiments. **a** Model architecture. From the previous model design, a group of 450 spatially modulated non-grid/LV cells is added to the EC cell population, the number of Grid cells and LV cells is reduced to 150 to achieve the EC ratio of Grid cells and non-grid cells reported^23^, and a subgroup of 300 GCs under weak inhibition is added to the GC population. The firing fields of non-grid/LV cells were generated by the multiplication of noise and Gaussian signals to achieve lower spatial information^23^. The color scale is the same as that used in (**b**). **b** Transformation of spatial representations across iterations. Color-coded representation of rate maps for active GCs under high (blue) and low (green) inhibition, across iterations. In gray, the emergence of non-grid/LV cells across iterations. **c** Upper, proportion of GCs (irrespective of type) with a single field (black) and multiple fields (gray), across iterations. Lower, proportion of LV, periodic and unspecific GCs, among multiple-field GCs, across iterations. Note the similarity with experimental trends (Fig. 3c). **d** The rate map of EC LV cells (upper), grid cells (middle) and non-grid/LV cells (lower) used to model the original (left), reordered (middle) and new (right) belts. To simulate the reordered belt, the firing fields of EC LV cells are moved to the new location of the landmarks, and 40% of non-grid/LV firing fields are randomly regenerated. To simulate the new belt, EC LV cells are randomly assigned to new landmarks, and all non-grid/LV firing fields are randomly regenerated. The color scale is the same as that used in (**b**). **e** Fraction of GCs with single (black) and multiple (gray) place fields in each belt, across iterations (*n* = 3300 cells). Note the similarity with experimental trends (Fig. 5b). **f** The distribution of the number of fields per cell (using the belt with the largest number of fields) for the groups of GCs that are active in 1 (black), 2 (green), and 3 (purple) belts, across iterations. Note the similarity with experimental trends (Fig. 5d).

The model did not, however, replicate the progressive emergence of multiple unspecific place fields. Reducing feedback inhibition increased the number of multiple-field cells, but these cells were mostly periodic or LV cells, and the overall fraction of cells generating place fields was excessive (Supplementary Fig. 6). Therefore, we reasoned that unspecific place fields should be generated by a small subset of GCs under low inhibition that receive excitatory inputs from cells showing no periodic and LV activity patterns. Such a subset of excitable GCs could correspond to the small population of young adult-born GCs, which are generally more excitable and active than mature GCs^45–48^. On the other hand, non-periodic/LV spatial inputs could be supplied by the large population of EC non-grid spatially modulated cells reported in familiar environments^23^. To test this hypothesis, we added a population of 300 GCs under weak feedback inhibition and a population of 450 non-grid/LV spatially modulated EC cells to the model (the numbers of grid cells and LV cells were each reduced to 150 such that grid cells represented 20% of the EC cells^23^, and the EC grid:LV ratio remained 1:1, a factor critical to reproduce the initial 1:1 ratio of periodic and LV GCs; Fig. 8a). However, to reproduce the initial predominance of periodic and LV activity patterns of GCs, the number of active non-grid/LV cells was set to zero initially and was then progressively increased over iterations (a plausible phenomenon considering learning within EC and the increasingly strong spatial input feedbacked from the hippocampus; Fig. 8b top). As anticipated, the subset of excitable GCs developed multiple unspecific place fields over time (Fig. 8b), and the model could largely reproduce the experimental trends for single, LV, periodic and unspecific cells (Fig. 8c).

We next examined the predicted cell activity for the reordered and new belts. To simulate the reordered belt, the firing fields of EC LV cells were moved to the new location of the landmarks, and 40% of the non-grid/LV cells were randomly assigned new activity patterns, whereas to simulate the new belt, EC LV were randomly assigned to new object pairs, and 100% of the non-grid/LV cells were randomly assigned new activity patterns (Fig. 8d). For both the reordered and new belts, the activity of grid cells was not altered to reflect the relative preservation of grid cell patterns across environments as opposed to the strong remapping of EC non-grid spatial cells^23^. Similar to the experimental data (Fig. 5b, d), the number of single-field representations was decreased for the reordered belt compared to the original belt and was further decreased for the new belt (Fig. 8e), and the number of place fields per cell was correlated with the number of belts represented (Fig. 8f). Hence, this version of the model could reproduce both the transformation of GC representations across days for the original belt and the subsequent encoding of the reordered and new belt layouts.

Importantly, an alternative model in which the subset of excitable GCs received excitatory inputs from the other GCs could also reproduce the diverse aspects of the experimental data (Supplementary Fig. 8). In this version of the model, EC non-grid/LV cells were not incorporated, and GC activity evolved solely from the synaptic plasticity processes. The excitable GCs generated initially periodic and LV firing fields from the sparse inputs of the other GCs and then, over time, developed multiple unspecific place fields reflecting combinations of the single place fields of the GCs. Such network configuration is plausible considering the report that mature GCs transiently provide synaptic inputs to young adult-born GC^49^ and thus might contribute to the emergence of GC multiple unspecific place fields.

## Discussion

Using silicon probe recording in a treadmill apparatus and neural network modeling, we identified putative GCs and MCs, monitored the development of spatial representations during learning of a particular layout of landmarks, investigated subsequent encodings of other layouts, and interpreted the data in terms of learning mechanisms, information encoded and cell-specific functions. Recent studies have outlined cell-type specific differences in the scale, sparseness, stability, and remapping of DG spatial representations. Our study provides data and mechanistic insights on how the spatial representations develop during learning, uniformly map the space and encode other similar environments.

Like our previous study^17^, we used the combination of spike autocorrelograms and spike relationships with gamma oscillations and a subset of opto-tagged DRD2 and POMC cells to segregate and identify putative GCs and MCs. Opto-tagged POMC cells may have been biased toward a relatively young subpopulation of GCs, as POMC expression is limited to the 1-month period following cell mitosis and opto-tagging experiments were carried out 4 weeks after virus injections^17^. However, in addition to the overlap with opto-tagged POMC cells, the cluster of putative GCs was suggested by the non-overlap with opto-tagged DRD2 cells and the fact that putative GCs were located above putative MCs along the electrode shanks. Our data showed both consistencies and discrepancies with previous reports. First, MCs were characterized by low burst activity, consistent with in vivo intracellular recording data^40^, but at odd with recent studies using distinct classification and opto-tagging strategies^13,14^, which could reflect differences in cell classification, animal behavioral states, and/or methods to assess burst activity. Second, GCs and MCs showed distinct gamma phase preferences and GCs were more modulated by gamma oscillations than MCs. While these effects were also observed in a recent study^14^, it is notable that the difference in spike gamma phase was larger in our study, which could reflect differences in cell classification, animal behavioral states, and/or electrode locations for gamma oscillation measurements. Third, as previously reported^12–14^, the fraction of active cells was lower for GCs than for MCs, and each GC preferentially exhibited a single place field once the belt layout was familiar (days 10–20) whereas MCs preferentially exhibited multiple place fields. Approximately two-thirds (64%) of the spatially modulated GCs exhibited a single place field on the familiar belt, a fraction similar to recent reports^13,14^ but larger than in a study where DG recordings were carried out in a progressively morphed arena^11^. While the lower fraction in that study might result from the lack of proper criteria to segregate GCs and MCs, another explanation could be the novelty component introduced by the progressive morphing of the arena, considering our observation that the proportion of single-field GCs was decreased in the new belt layouts. Last, like previous reports, the GCs that exhibited single and multiple place fields tended to represent single and multiple environments, respectively^13,47^.

We reported diverse types of firing fields in the DG and a strong effect of experience on the incidence of each type. In particular, LV and periodic cells were not reported previously, and our findings suggest that their incidence largely increases in novel environments. It should be mentioned, however, that these two types of representation might also be enhanced by the oversimplification of sensory information in the treadmill^35^ and that their occurrence in typical maze environments might be rare. Importantly, we observed a progressive transformation of GC spatial representations over the course of several days, characterized by an asymptotic increase in the number of place cells, a conversion in place field types, and decreases in place field emergence and extinction rates. Another study that carried out long-term recordings of hippocampal spatial activity has emphasized a relatively high stability of GC spatial representations^15^; however, recordings were started after >10 days of habituation in their first environment tested (by that time, the development of GC spatial representations almost reached a plateau in our study), and a progressive development of GC spatial correlations was visible in the second (novel) environment tested, consistent with the slow dynamics we observed. Furthermore, the number of GCs exhibiting place fields was proportionally reduced in the reordered and novel belts according to the level of similarity with the original belt. A similar reduction in GC place cells was previously observed^15^, but the impact of context similarity was not tested. It is notable that the extent of GC remapping largely differs among studies^11–15,17,47^, a discrepancy that is probably explained by the diverse types of environments and cue alterations involved (open arena^11,13,14^, virtual linear track^15^ or treadmill^12,17,47^ environments; changing the room^13^, the layout of cues^12,15,47^, the arena boundaries^11^ or some local cues^14,17^). In the treadmill, the landmarks fixed on the belt provided the only environmental information useful for mapping positions on the belt, and altering the layout of landmarks is expected to have a larger impact than, for instance, altering local cues in an open arena^14^ where unchanged spatial information is supplied by distal cues. Last, we observed that MCs, but not GCs, overrepresented the landmarks of the belt. This observation supports the idea of a weak coupling between MC and GC activity, consistent with the weak GC-to-MC spike transmission^14^ and the distinct MC and GC responses to local cue manipulations^14,17^, but raises questions regarding the potential origin of the increase of MC activity in landmark locations. Apart from the GCs, MCs receive external inputs from CA3, semilunar GCs and EC cells^26,27^. The few putative CA3 cells we monitored did not show much increased activity in the landmarks, making CA3 inputs an unlikely mechanism. On the other hand, semilunar GCs are especially responsive to performant path stimulations and generate large barrages of excitatory postsynaptic potentials in MCs^26^. Together with direct EC-to-MC inputs, they could relay to MCs a strong landmark-modulated signal.

Our findings suggest that competitive learning underlies the increase in spatial representations in DG. The model reproduced the transformation and the asymptotic increase in GC representations across days, as well as the subsequent encoding of other belt layouts. How place fields initially emerge through competitive learning is not straightforward, as cells that are silent paradoxically develop place fields as a result of Hebbian synaptic plasticity, which requires postsynaptic firing. In the model, the initial activation of silent GCs could only be elicited through disinhibition, which had to result from a local decrease in the mean GC activity following the synaptic normalization operation. Hence, disinhibition induced by synaptic normalization might be the initial trigger for the generation of GC place fields via competitive learning. Hebbian synaptic plasticity might then be involved to strengthen EC inputs to GCs in the place field locations. The involvement of a Hebbian form of synaptic potentiation is suggested by the observation that GCs receiving electrical stimulations tend to develop place fields in the stimulus locations^50^. Note that a form of synaptic plasticity depending less on pre and postsynaptic coincident activity and more on non-linear dendritic response dynamics is believed to take place in CA1 (refs. ^51–53^). Over the days, the number of GC place cells progressively plateaued and the rates of place field emergences and extinctions were progressively decreased, a phenomenon reminiscent of the reduced likelihood for electrical stimuli^50^ and head scanning movement^54^ to generate place fields as environments become familiar. In the model, this phenomenon was correlated with a progressive reduction of synaptic changes, as the synaptic weight matrix converged to a state where the effect of synaptic potentiation was matched and reversed by the effect of synaptic normalization. Likewise, the observed plateauing of the GC development might correspond to such equilibrium in synaptic plasticity mechanisms. Finally, increasing inhibition in landmark locations was critical to reproduce the uniform mapping of the space by GCs. In general, a close match between feedforward inhibition and average input activity was an effective mechanism to achieve uniform mapping via competitive learning, suggesting that it might be a recurrent feature of brain networks. MCs are well suited to support such an operation, as they showed firing rate increases in landmark locations and generate feedforward inhibition in GCs^27–33^. Hence, we suggest that one function of MCs is to sense variation in EC input via semilunar GCs and/or direct EC afferents^26,27,29^ and proportionally increase inhibition to ensure a uniform mapping of the space by GCs.

Interestingly, the development of GC spatial representations was relatively slow, plateauing after a week. Periods of rest between treadmill running sessions were likely essential to regenerate the network potency for plasticity, as the place field emergence rate progressively decreased across trials within each session (Fig. 4d). Synaptic normalization, in particular, might require sleep and sharp-wave-ripple oscillations^55,56^, considering the relatively slow rate of synaptic downscaling observed in vitro^44^. Such slow learning is in theory critical for minimizing the alteration of prior memories by new learning (the stability-plasticity dilemma^57^), is consistent with the high stability of DG spatial representations over time^15^, and should make DG information storage more specific to stable features of the environment and therefore particularly suitable for a reliable encoding of spatial contexts. However, it seems at odds with the fast encoding processes typically associated with episodic memory that allow for instance to recall object locations after a quick exploration of a novel environment. This has several possible interpretations. First, spatial learning might be slower in the treadmill because of the relative sparsity of cues and the restriction of head movements^54^. In this respect, it is likely that learning dynamics also vary in natural environments depending on the abundance of cues and arousal states. Second, it is possible that fast encoding mostly takes place in the CA regions^15^, whereas the DG contributes to episodic memory mainly by supplying a reliable spatial context information and helping differentiate CA3 encodings via pattern separation^6–10^. Last, it should be pointed out that the plateauing of the GC development corresponded only to an optimum in information storage, in which a long time delay to reach the plateau might not be incompatible with the long time delay required to perfectly know an environment (for instance, it might take weeks to become familiar with the sequence of stores and restaurants along a street), and does not prevent that information storage occurring during single trials or days before the plateauing might already be enough to support some approximative memory of the belt (street) layout.

The conversion in place field types across days suggests the integration of diverse information. As we implemented in the model, LV and periodic GCs might be generated by strong excitatory inputs from LV cells and grid cells in the EC, respectively. While grid cells are typically characterized by a two-dimensional triangular grid arrangement of firing fields, they noticeably exhibited linear periodic patterns in the one-dimensional alleys of a hair-pin maze^58^. Such periodic activity pattern, so far unseen in the hippocampus, may have emerged in the treadmill because of the novelty of the environment (considering that periodic cells almost completely disappeared after day 1), the oversimplification of the spatial information, and/or the fact that some of the landmarks on the belt were periodically interspaced (Note the periodic arrangement of every second landmarks). A possible scenario is that the periodic GCs originated from the combination of landmark-vector mechanisms and grid cell spatial distance information, the latter accounting for the selection of encoded landmarks based on landmark spatial periodicity rather than identity. The model largely implied an integration of LV cell and spatial cell inputs, such that GC activity was contingent upon the particular alignment of object and spatial information on the belt. Such integration might underlie the elaboration of memory engrams for spatial context^15,59^ that incorporate object location information. Through such engrams, GCs would produce an output very specific to the spatial context and would remap when the object layout is changed^17^, consistent with the importance of an intact DG to discriminate contexts and detect changes in object layouts^8,20,60^. DG function might also be considered in terms of pattern separation, with the combination of context engram, sparse GC activity and inter-GC competition contributing to strong remapping responses by GCs^17^. In this respect, our findings imply that pattern separation should improve with experience as context engrams progressively develop, whereas pattern separation in a new context should be enhanced by prior experience in other similar contexts, given the generalization of learning across similar belt layouts.

Reducing the model inhibition threshold for a small group of GCs was critical to reproduce the emergence of unspecific GCs, suggesting that multiple unspecific firing fields were generated by more excitable GCs, possibly the young adult-born GCs that are known to exhibit multiple firing fields, low context specificity and high levels of excitability^45–48^. Another requirement to reproduce the emergence of unspecific GCs was a progressive increase of excitatory inputs from cells exhibiting non-grid/LV activity patterns. This could be achieved either by adding a population of EC cells that progressively developed spatially modulated non-grid/LV firing fields^23^ or by using the main GC population as input to the subpopulation of excitable GCs. Both mechanisms are plausible considering potential learning processes within the EC, the increasingly strong spatial input feedbacked from the hippocampus, and reports that young adult-born GCs initially receive inputs mostly from MCs, the CA3 and mature GCs^48,49^. While the coexistence of less-excitable and more-excitable GCs could enable a differential encoding of the familiar and unique features of contexts (Fig. 5b), another possible advantage is that it allows parallel operations requiring low and high cell excitability, such as pattern separation and temporal binding of episodic information^61^, respectively. In this respect, it is noteworthy that the network configuration shown in Supplementary Fig. 7 could allow a temporal binding operation by young adult-born GCs that does not compromise DG pattern separation, as young adult-born GCs relay the strongly differentiated information from mature GCs.

In conclusion, our findings suggest that a slow integration of spatial cell and landmark-vector cell inputs, achieved via competitive learning, is the mechanism underlying both the emergence of spatial representations and the continuous mapping of the space by GCs, while a function of MCs is to ensure a uniform distribution of GC representations. As a result, the DG may generate stable maps of the environments that embed object information, allowing the discrimination of slightly different contexts and the detection of slight changes in object layouts. Further work may test whether competitive learning mechanisms operate on the whole DG network or on the level of DG subnetworks associated with distinct information, time scales and cell morphology^62^.

## Methods

### Animals

All experiments were conducted in accordance with institutional regulations (Institutional Animal Care and Use Committee of the Korea Institute of Science and Technology) and conformed to the Guide for the Care and Use of Laboratory Animals (NRC 2011). Four male C57BL6 mice between 6 and 7 weeks old were used. The mice were housed 2 per cage in a vivarium under a 12 h light/dark cycle.

### Virus injection and preparation for head fixation

During a first surgery, under isoflurane anesthesia (supplemented by subcutaneous injections of buprenorphine 0.1 mg/kg, followed by daily subcutaneous injection of ketaprofen 5 mg/kg for 2 days), two small watch screws were driven into the bone above the cerebellum to serve as reference and ground electrodes for the recordings. A 3D printed plastic head-plate with a window opening in the center was cemented to the skull with dental acrylic. The head-plate was designed to be conveniently fixed/unfixed to a holding plate using two screws.

### Treadmill apparatus and behavioral training

After a post-surgery recovery period of 7 days, the mice were water restricted to 1 ml of water per day and were trained for 2 weeks (one 1-h session per day) to run on the treadmill with their heads fixed. The treadmill was not motorized but consisted of a light velvet belt laying on two 3D printed wheels, which mice moved themselves at will. Water rewards were delivered on every trial at the same position on the belt (position 0 cm) via a lick port. After behavioral learning reached an asymptote, the animals completed 100 to 150 trials in the first 45 min of each session. The quantity of water consumed on the treadmill was measured after each session, and additional water was provided such that the mice drank a total amount of 1 ml of water per day.

For recording sessions, landmarks were fixed and interspersed on a 201-cm long belt. The landmarks consisted of arrays of vertical poles and textures fixed on the edges of the belt, which provided visual-tactile stimulation to both sides of the mice (Supplementary Fig. 4).

The treadmill presents several advantages. First, the animal behavior and trajectory across landmarks are very consistent over trials and sessions. This aspect is important to reliably measure changes associated with learning. Second, the spatial information is simpler and well controlled. Indeed, the landmarks fixed on the belt provide the only useful environmental information for mapping the positions of the belt and are experienced one at a time. As a result, we could isolate distinct spatial mechanisms (LV, periodic, unspecific, single) and observe a transformation of spatial representations. Also, modeling the experiment was straightforward. A downside of the treadmill is that some exploratory behaviors (like head scanning movements) are prevented by the head fixation. Because distal cues are missing, the treadmill belt environment might be similar to channels in high grass or underground where mice should rely mostly on local cues and path integration.

### Chronic implantation of the electrode

A 64-channel silicon probe (Neuronexus, Buzsaki64sp) was used for chronic recordings. A craniotomy (1.5 mm long and 0.5 mm wide) was performed above the hippocampus under isoflurane anesthesia^35,36^. The silicon probe was mounted on a custom microdrive^63^, coated with red-fluorescent dye (DiI, Life technologies), and lowered into the granule cell layer of the DG, which was detected by the emergence of unit activity following a ~500-µm zone of unit activity silence below the CA1 pyramidal layer. The microdrive was then cemented to the skull and head-plate. A bone wax and mineral oil mixture was used to cover the craniotomy. A plastic cap was used to protect the microdrive/silicon probe assembly^63^.

### Anatomy

On the last day of recording, the animals were anesthetized at the end of the recording and perfused transcardially with 4% paraformaldehyde in phosphate buffer. The brain was removed and kept overnight in 4% paraformaldehyde solution. Coronal sections (100 µm thick) were sliced using a vibratome and were mounted on slides using Vectashield mounting medium with DAPI. Images of DAPI and DiI fluorescence were acquired separately with a Nikon FN1 microscope equipped for fluorescence imaging. The DG and the electrode signals were isolated and visualized in 3D using custom MATLAB routines.

### Behavioral control and data acquisition

The forward and backward movement increments of the treadmill were monitored using two pairs of LEDs and photosensors that read patterns on a disc coupled to the treadmill wheel, while the zero position was implemented by an LED and photosensor coupling that detected a small hole on the belt. From these signals, the mouse position was implemented in real time by an Arduino board (Arduino Uno, arduino.cc), which also controlled the valves for the reward delivery. Position, time and reward information from the Arduino board was sent via USB serial communication to a computer and recorded with custom LabView (National Instruments) programs.

Neurophysiological signals were continuously acquired at 30 kHz on a 250-channel recording system (Intan Technologies, RHD2132 amplifier board with RHD2000 USB Interface Board and a custom LabView interface). The wideband signals were digitally high-pass filtered (0.8–5 kHz) offline for spike detection and low-pass filtered (0–500 Hz) and downsampled to 1000 Hz to extract local field potentials. Spikes from each session and each shank of the silicon probe were clustered separately with automatic algorithms^37^ followed by manual adjustments in custom MATLAB routines implementing spike autocorrelation, cross-correlation and cluster isolation statistics. Only clusters with well-defined cluster boundaries and clear refractory periods were included in the analyses^38^.

### Estimation of cell position relative to the shank

To estimate the position of a cell relative to the recording sites on a shank, we assumed that the amplitude of the spike signals are attenuated as 1/*d*^2^ (see note below), where *d* is the distance of the site to the cell soma, such that the amplitude measured at a given site is:

$$a_i = A/d_i^2$$

with *A* being the spike amplitude at the cell position.

For the many recording sites on one shank, this means that:

$$A = a_1\ast d_1^2 = a_2 \ast d_2^2 = a_3 \ast d_3^2 = a_4 \ast d_4^2 = a_5 \ast d_5^2.$$

Therefore, to estimate the position of a cell, we simply searched for the position at which these conditions were fulfilled. To do this, the volume around each shank was divided into 1 µm^3^ pixels, and for each pixel, we computed the Euclidean distances to each recording site. Then, we defined a value *S* such that

$$S = \sum _{ij}|a_i \ast d_i^2 - a_j \ast d_j^2|,$$

where *i* and *j* varied to generate all possible combinations of sites.

The pixel with the smallest value of *S* was defined as the cell position.

Note: Electric potential of dipoles are attenuated as 1/*d*^2^ but as 1/*d* for monopoles. We tested this method using either form and found that the resulting cell positions was very similar.

### Spike gamma phase

The LFP from a channel in the hilus was bandpass filtered between 30 and 80 Hz. A vector of instantaneous phase was derived using the Hilbert transform. The gamma phase of each spike was interpolated from the vector of the instantaneous phase.

### Gamma coupling index

The ‘gamma coupling index’ captures the coupling of LFP gamma power to cell activity during animal immobility. For each cell, the average gamma power of a hilar LFP was computed for windows within (−10 to +10 ms) and outside (+40 to +100 ms) epochs of maximal firing activity, and the gamma coupling index was defined as the difference between the two windows divided by the sum of the two windows. The 100 largest peaks of the smoothed (using a 20 ms half-width Gaussian kernel) instantaneous firing rate vector were used as epochs of maximal firing.

### Implementation of single neuron firing rate vector

The length of the belt was divided into 100 pixels. To generate a vector of firing rates, the number of spikes discharged in each pixel was divided by the time the animal spent in the pixel. The firing rate vector was smoothed by convolving a Gaussian function (15 cm half-height width).

### Place field emergence and extinction

For a given place field, the mean firing rate in a 10-cm window enclosing the field was calculated for each trial, producing a vector of firing rates. The vector was smoothed using a 3-trial half-width Gaussian kernel. The emergence of a place field corresponded to the time point when the firing rates increased and exceeded 20% of the vector peak value, while the extinction of a field corresponded to a decrease below 20% of the vector peak value.

### Statistics and reproducibility

All statistical analyses were performed in MATLAB (MathWorks). The number of animals and the number of recorded cells were similar to those generally employed in the field. The analysis of variance (ANOVA) was used to test mean differences between groups larger than two. Student’s *t* tests were used to test the sample mean. Correlations were computed using Pearson’s correlation coefficient.

### Reporting summary

Further information on research design is available in the Nature Research Life Sciences Reporting Summary linked to this article.

## Supplementary information

Supplementary Figures

Peer Review File

Reporting Summary

## Data Availability

Due to their large size, the data collected for this study are available upon reasonable request to S.K. or S.R. Source data are provided with this paper.

## Source data

Source Data

## Code availability

All the codes for data analysis and modeling are available upon reasonable request.
