# Supplementary material for: Place cell maps slowly develop via competitive learning and conjunctive coding in the dentate gyrus
Source: Nat Commun. 2020 Sep 11;11:4550. doi: 10.1038/s41467-020-18351-6 (PMC7486408; doi:10.1038/s41467-020-18351-6)
Supplement: Supplementary file 1 — Supplementary Figures [file 41467_2020_18351_MOESM1_ESM.pdf]

Supplementary Information

Place cell maps slowly develop via competitive learning and conjunctive coding in the dentate gyrus

Kim et al.

Supplementary Figure 1

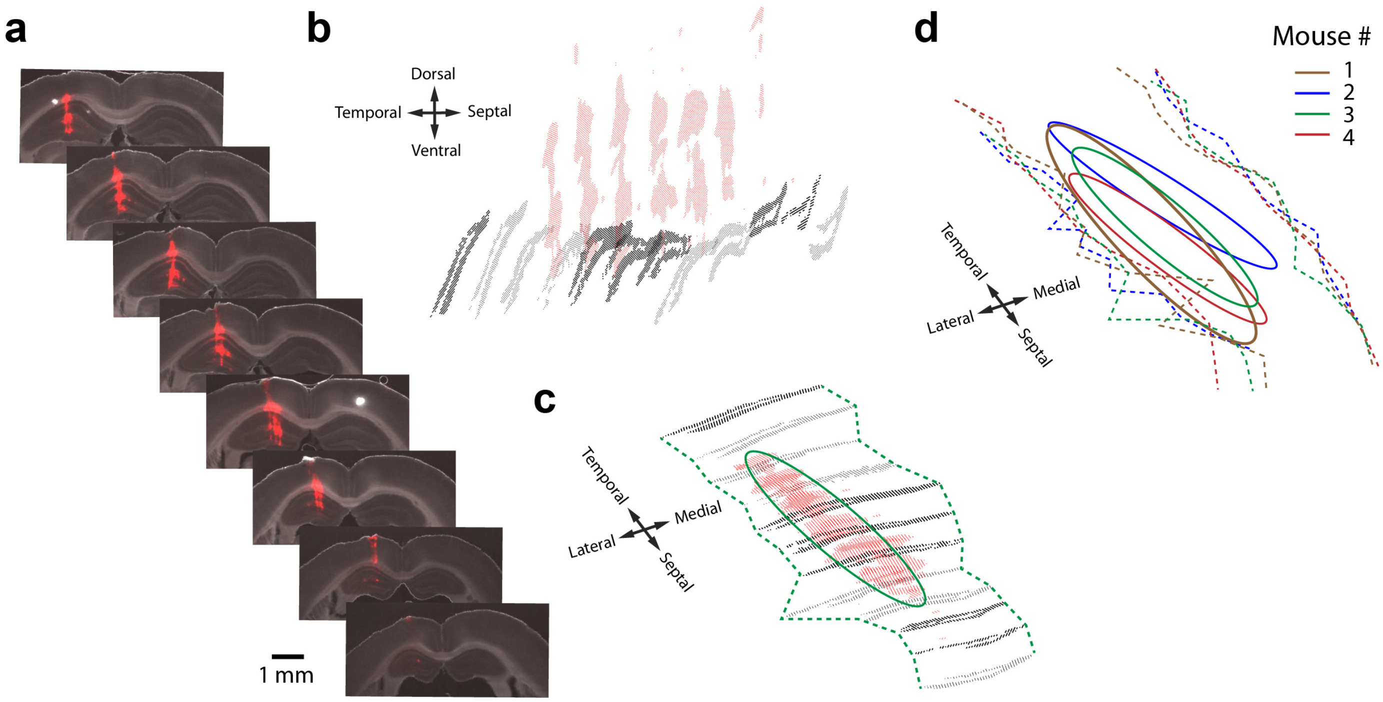

Supplementary Figure 1. Histology and electrode positions

a) Example of coronal sections for one animal showing the tracks of the electrodes (*red*, DiI). Images reconstructed by overlaying DAPI and DiI fluorescence images.

b) Granule cell layers (black dots) and electrode tracks (*red dots*) were detected for each coronal section using custom MATLAB routines, and 3D reconstructions were implemented for each mouse.

c) The 3D images were rotated perpendicular to the electrode tracks to estimate the electrode positions (*ellipsoid*) relative to the lateral/medial edges of the granule cell layer (*dashed lines*).

d) Summary diagram of electrode positions for all mice.

Supplementary Figure 2

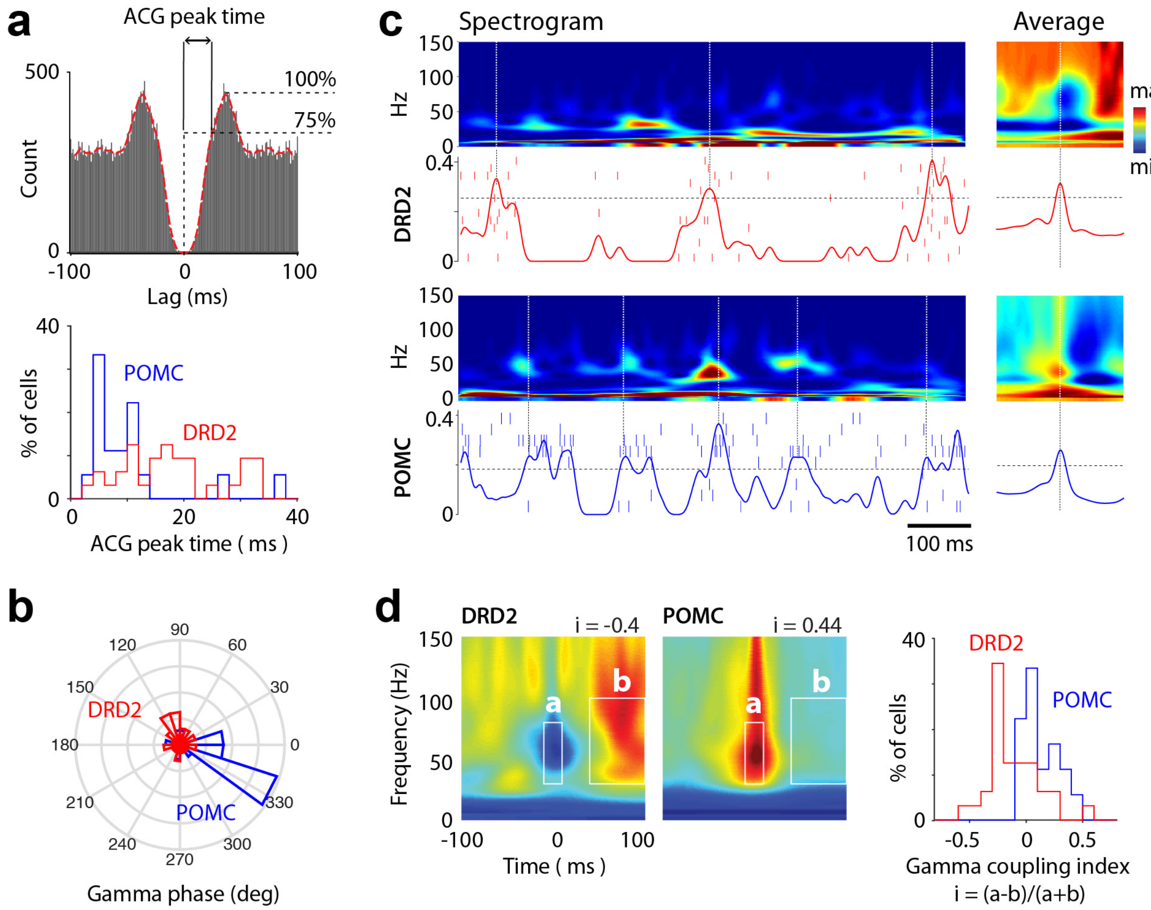

Supplementary Figure 2. Spike features of light-excited cells in DRD2 and POMC mice

a) Upper, ACG of a cell example and illustration of the ACG refractory gap measure (defined as the duration for spike ACGs to reach 75% of peak value). Lower, distribution of ACG refractory gap for light-excited cells in DRD2 (red) and POMC (blue) mice. Spike ACGs were characteristic of short interval burst activity for POMC light-excited cells and showed large humps flanking a wide refractory gap in DRD2 light-excited cells, consistent with GC and MC ACGs observed during in vivo intracellular recordings.

b) Distribution of the mean gamma phase for DRD2 (red) and POMC (blue) light-excited cells. Note that POMC light-excited cells show a strong preference to discharge near the troughs of gamma oscillations while DRD2 light-excited cells show a slight preference to the opposite phase.

c) Coupling of cell activity with gamma power. Spectrograms (color-coded), spike rasters of light-excited cells (ticks) and average instant firing rate of light-excited cells (lines), for one DRD2 (upper) and one POMC mouse (lower). Events of strong population activity are detected on the instant firing rates (using a threshold equal to 10% of the largest event, dash lines, left) to generate event-triggered spectrogram averages (right). Note that the activity of light-excited cells is inversely correlated with gamma power for DRD2 and POMC.

d) Gamma coupling indexes of individual cells. Left, spike-triggered spectrogram averages for one DRD2 and one POMC light-excited cell. To compute the gamma coupling index i, the mean power in regions a (-10 to 10 ms, 30 to 80 Hz) and b (30 to 100 ms, 30 to 100 Hz) were calculated, and then i = (a-b)/(a+b). Right, distribution of gamma coupling indexes for DRD2 and POMC light-excited cells. Note that spiking activity of is positively coupled with gamma power for POMC light-excited cells while negatively coupled for DRD2 light-excited cells.

Supplementary Figure 3

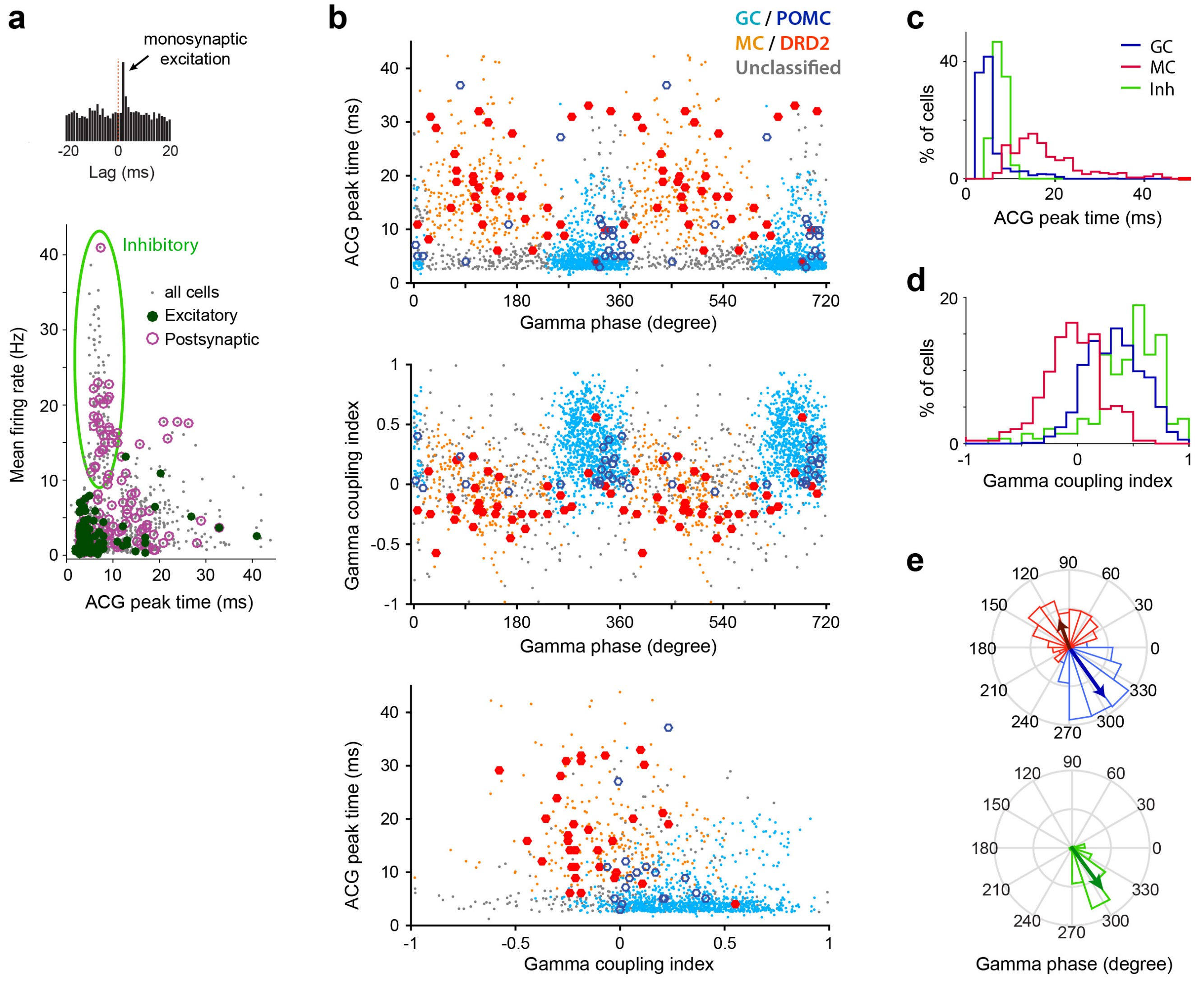

Supplementary Figure 3. Identification of putative GCs and MCs

a) Mean firing rate (during animal immobility) versus ACG refractory gap for all cells (gray) and a subset of cells identified as presynaptic excitatory (green) and postsynaptic (purple) cells from a large peak at monosynaptic latency <3 ms in the cross-correlogram of a neuron pair (inset). Green ellipsoid, putative inhibitory interneurons segregated by high firing rate, short ACG refractory gap and lack of identified excitatory neurons.

b) Scatter plots for spike gamma phase, ACG refractory gap and gamma coupling index of all cells, except the putative inhibitory cells identified in (A). Putative MCs (orange dots) and GCs (light blue dots) are identified by overlap with DRD2 (red filled circle) and POMC (blue circle) light-excited cells.

c-e). Distribution of ACG refractory gap (c), gamma coupling index (d) and spike gamma phase (e) for putative GCs (blue), MCs (red) and inhibitory cells (green).

Supplementary Figure 4

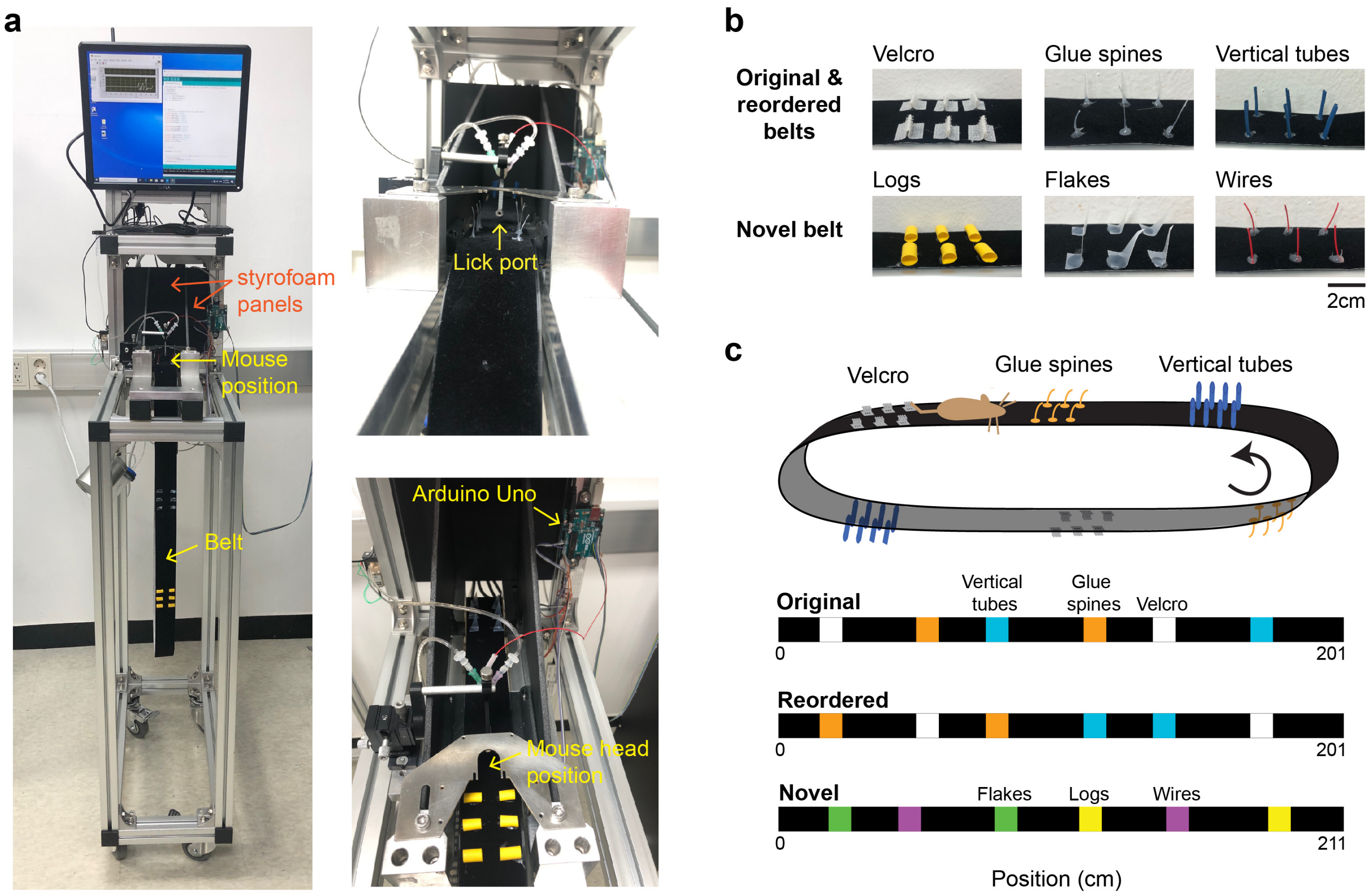

Supplementary Figure 4. Treadmill apparatus and belt layouts

a) Pictures of the treadmill apparatus.
b) Pictures of the different types of landmarks fixed on the original, reordered and novel belts.
c) Layout of the landmarks on the original, reordered and novel belts.

Supplementary Figure 5

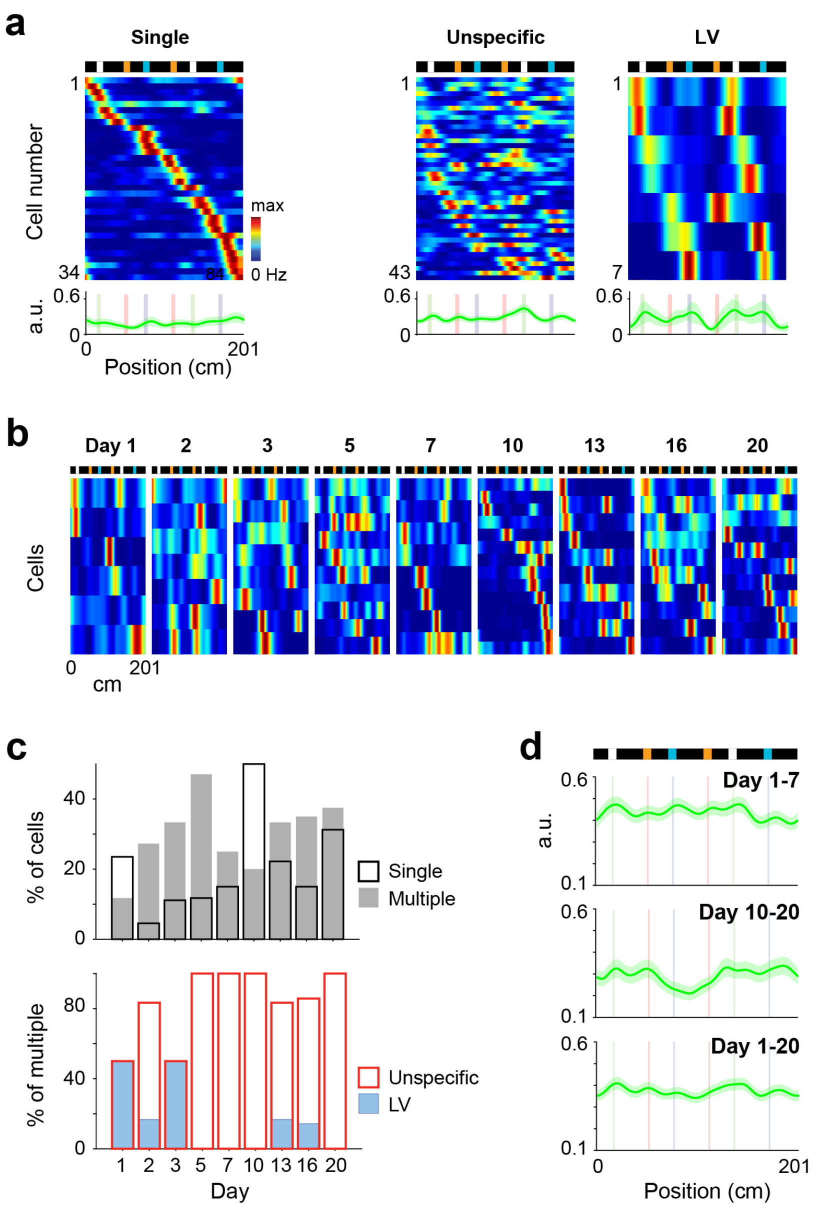

Supplementary Figure 5. Spatial representation of putative CA3 cells

a) Color-coded, firing rate maps of putative CA3 cells from all sessions, sorted according to field positions and grouped by type of representation. Line plots, average of firing rate maps for each type of representation (lines, the mean; shadows, s.e.m; n = 34 single, 43 unspecific and 7 LV cells).

b) Color-coded, firing rate maps of putative CA3 cells across days. Only the cells showing firing fields are displayed for clarity. The rows of the matrices correspond to individual cells and are sorted according to firing field positions. Top, scheme of the belt.

c) Upper, proportion of cells with a single field (black, n = 34 cells) and multiple fields (gray, n = 50 cells) across days. Lower, proportion of LV and unspecific cells, among multiple field cells, across days.

d) Average of rate maps (line, the mean; shadow, s.e.m) for days 1-7 (top, n = 94 cells), days 10-20 (middle, n = 74 cells) and days 1-20 (bottom, n = 168 cells).

Supplementary Figure 6

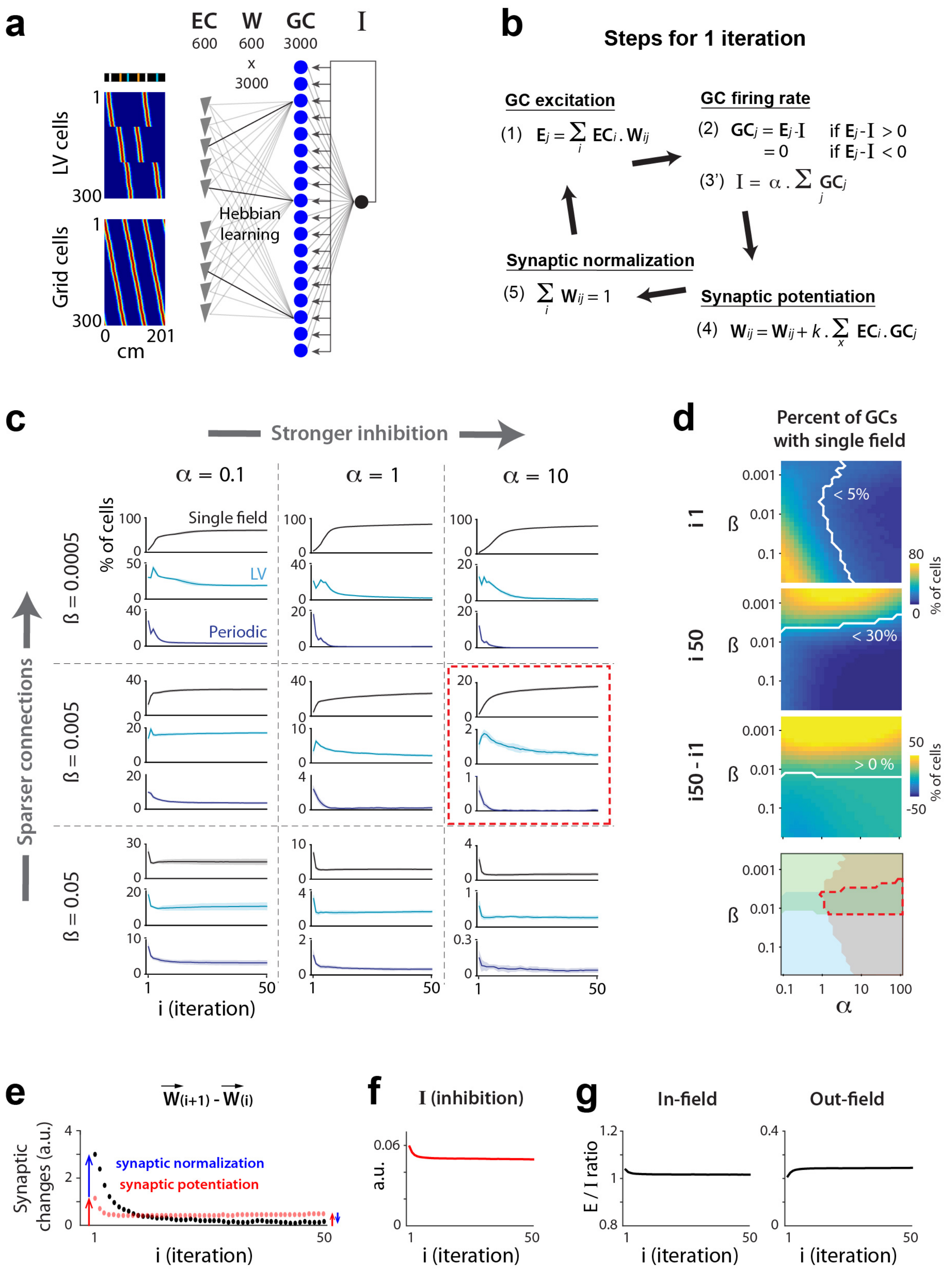

Supplementary Figure 6. Competitive learning reproduces the increase in GC single field representations

a) Model architecture. A total of 3000 GCs receive excitatory inputs from 300 LV cells and 300 grid cells from the EC, and are subjected to feedback inhibition. The EC-to-GC synaptic weight matrix is referred to as W_{ij}. The threshold used as feedback inhibition is referred to as I. Color coded, rate maps of EC LV cells (upper) and grid cells (lower). LV cells encode various distances to landmarks, while grid cells have various spatial phases and the same periodicity as periodic cells.

b) All operations were executed during one model iteration. 1) The excitation E_j received by the GC_j in a given position. 2) The levels of GC_j activation and feedback inhibition I in that position. The value of I is estimated numerically by finding the value among a range of I values that best satisfies the two relations. 3) The potentiation of synaptic weights, proportional to the level of EC-GC co-firing throughout the belt. 4) The normalization of synaptic weights.

c) Proportion of single (black), LV (light blue) and periodic (dark blue) firing field representations, across iterations, for different values of the parameters alpha and beta, used respectively to adjust the strength of inhibition and the initial sparsity of connections. The red rectangle indicates the conditions that best reproduce the experimental findings.

d) Top, color-coded representation of the proportion of GCs exhibiting a single place field in the first model iteration (i1), for the 28 alpha and 28 beta values tested. The pixels on the right of the white line have proportions <5% and are considered compatible with the experimental data. Second from top, the same as above for the 50th model iteration (i50). The pixels below the white line have proportions <30% and are considered compatible with the experimental data. Third from top, the difference between the 2 matrices above (i50-i1). The pixels above the white line have values >0 (i.e., single fields are increased) and are considered compatible with the experimental data. Bottom, overlay of the compatible zones defined above. The region of overlap between the zones (red dashed line) provides the possible alpha/beta values to reproduce experimental trends.

e) Change in synaptic weights following the synaptic potentiation (red) and synaptic normalization (black) operations, across iterations. The Euclidean distance $[\sum (W_{ij}(i+1) - W_{ij}(i))^2]^{1/2}$ was computed to measure change in the synaptic weight matrix W_{ij}. The red and blue arrows indicate the respective contributions of synaptic potentiation and synaptic normalization for the first and last iterations. Notice that synaptic changes exponentially decrease to near zero (explaining the plateauing of the single-field emergence) as the effect of synaptic normalization progressively matches and reverses the effect of synaptic potentiation.

f) Inhibition threshold, averaged over belt positions, across iterations. Notice that the inhibition is not increased, excluding such a mechanism for the plateauing of the single-field emergence.

g) E/I ratio (excitation over inhibition) averaged over cells and belt positions within (left) and outside (right) the place fields. Notice that the out-field E/I is not decreased, excluding such a mechanism for the plateauing of the single-field emergence.

Supplementary Figure 7

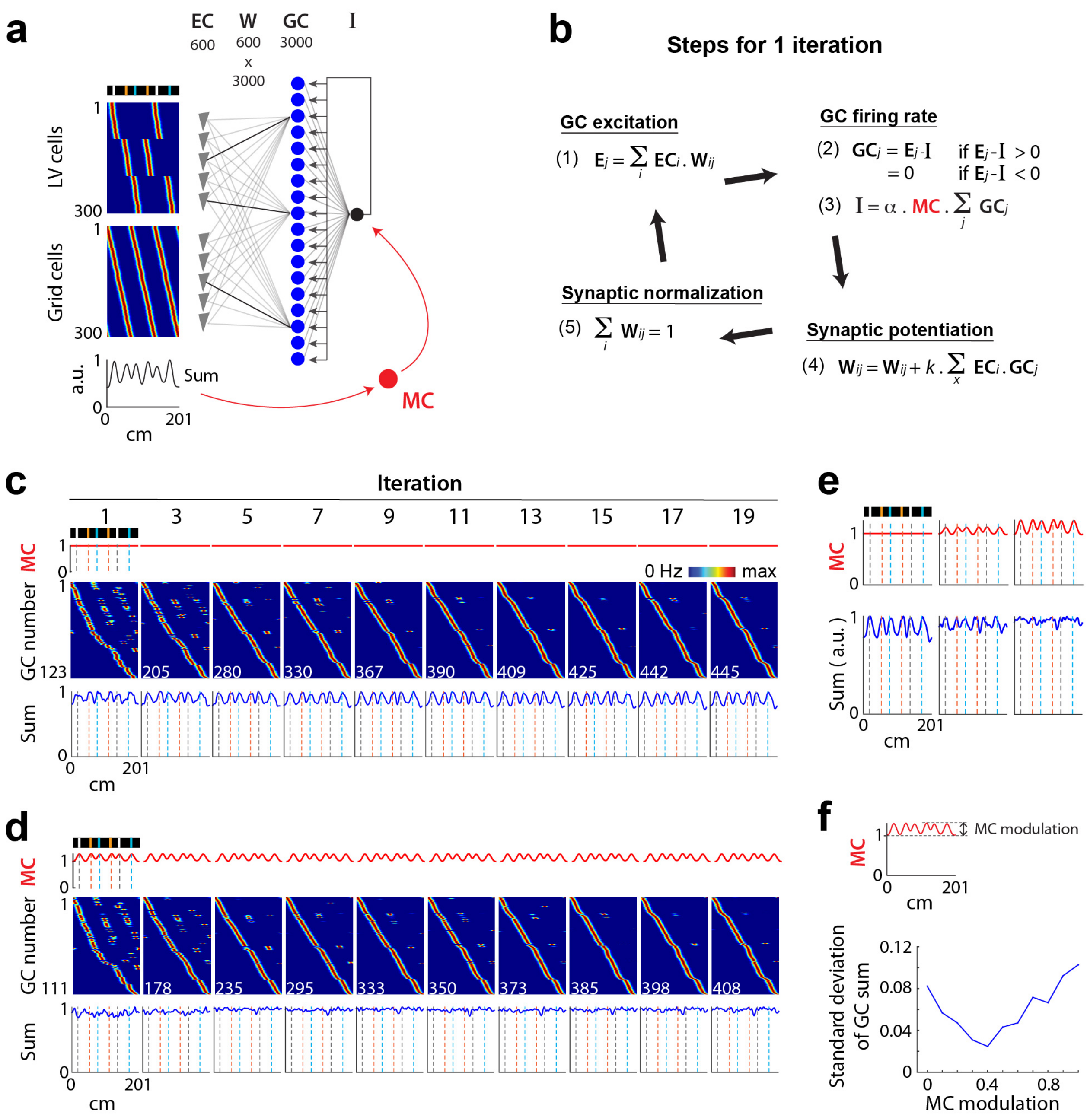

Supplementary Figure 7. Modeling the contribution of MC feedforward inhibition

a) Model architecture. MC feedforward inhibition is added to the previous model. The spatial modulation of MC activity is assumed to be proportional to the dynamic range of EC average activity.

b) All operations were executed during one model iteration. The only difference between this model and the previous one is that the strength of the inhibition is modulated throughout space by the factor MC.

c) In the absence of MC modulation, the average activity of GCs is increased in landmark positions. MC activity (top), GC rate maps (color-coded) and the mean of GC rate maps (bottom), across iterations.

d) With MC modulation, the average activity of GCs is uniform throughout the belt. The same format as c.

e) Effect of the distinct magnitude of MC modulation on the GC mean activity.

f) Spatial fluctuation in the GC mean activity as a function of MC modulation.

Supplementary Figure 8

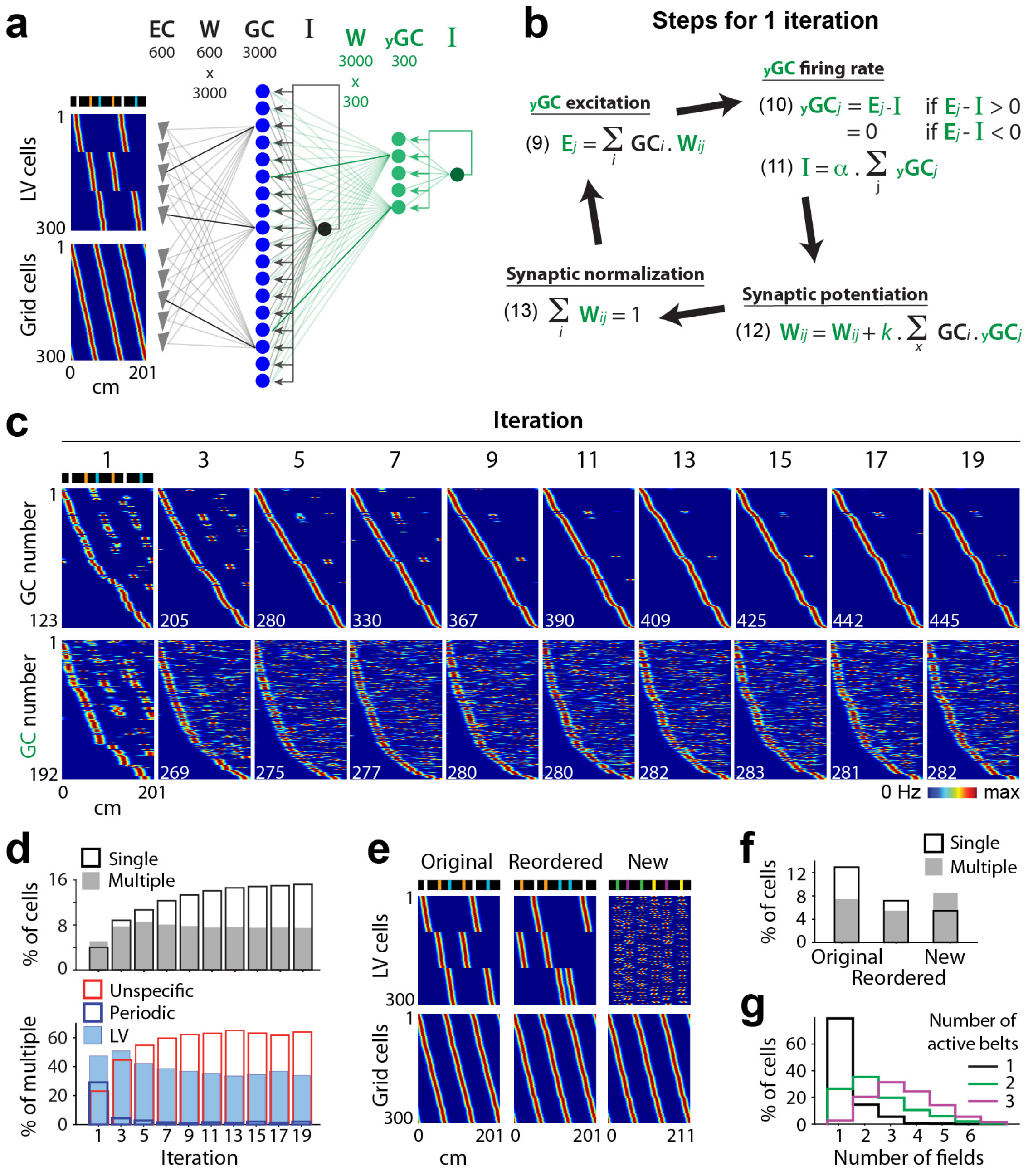

Supplementary Figure 8. Modeling of GC multiple unspecific firing fields

a) Model architecture. Three hundred 'immature' GCs (green) that receive inputs from the 3000 'mature' GCs and that are subjected to a low level of feedback inhibition are added to the previous model.

b) The same operations as those for mature GCs are executed for immature GCs in each iteration.

c) Color-coded representation of rate maps for active mature GCs (upper) and active immature GCs (lower), across iterations. Active cells have a mean activity > 0 and are sorted according to firing field positions. Note that immature GC representations initially resemble mature GC representations and then become multiple unspecific fields.

d) Upper, proportion of GCs (irrespective of type) with a single field (black) and multiple fields (grey), across iterations. Lower, proportion of LV, periodic and unspecific GCs, among multiple field GCs, across iterations. Note the similarity with experimental trends (Figure 1c).
e) The rate map of EC LV cells (upper) and grid cells (lower) used to model the original (left), reordered (middle) and new (right) belts. To simulate the reordered belt, the firing fields of EC LV cells are moved to the new location of the landmarks. To simulate the new belt, EC LV and grid cells are randomly assigned to new landmarks and spatial grid phases, respectively.
f) Fraction of GCs with a single (black) and multiple (gray) place fields in each belt. Note the similarity with experimental trends (Figure 3b).
g) The distribution of the number of fields per cell (using the belt with the largest number of fields) for the groups of GCs that are active in 1 (black), 2 (green) and 3 (purple) belts. Note the similarity with experimental trends (Figure 3d).
