# Supplementary material for: Place cell maps slowly develop via competitive learning and conjunctive coding in the dentate gyrus
Source: Nat Commun. 2020 Sep 11;11:4550. doi: 10.1038/s41467-020-18351-6 (PMC7486408; doi:10.1038/s41467-020-18351-6)
Supplement: Supplementary file 2 — Peer Review File [file 41467_2020_18351_MOESM2_ESM.docx]

Peer Review Information

**Journal:** Nature Communications

**Manuscript title: Place cell maps slowly develop via competitive learning and conjunctive coding in the dentate gyrus**

**Corresponding author name(s):** Dr Sebastien Royer

**Editorial notes:**

**none**

**Reviewer comments & decisions:**

**Reviewer comments, first version:**

Reviewers' comments:

Reviewer #1 (Remarks to the Author):

1. This is an interesting test of the theory of how competitive learning in DG sets up place cell activity from grid cell activity in EC.

The following comments are provided as feedback to help the authors improve the paper.

2. Line 73: "How such activity patterns emerge in the DG, the first processing stage of the hippocampal trisynaptic loop, is a fundamental question largely unresolved."

This could be stated more correctly as follows:
"How such activity patterns emerge in the DG, the first processing stage of the hippocampal trisynaptic loop, is a fundamental question. There is a clear theory that competitive learning in the DG and also at other stages of the hippocampus implements the transform (Rolls et al 2006; Si and Treves 2009), but empirical tests of the theory are still needed).

3. "Second, GCs are thought to operate as a competitive network in which strong feedback inhibition favors a ‘winner-take-all’ process and allows only a few GCs to be simultaneously active22,23."

The "winner-take-all' statement is not how the theory is held to apply to the brain, as it is most unlikely that neuronal systems ever produce a single winner, and that is not part of the theory. Nor does competitive learning involved only inhibition: it also involves synaptic modification, learning. I expect that the authors are fully aware of this, and the statement would be greatly improved by wording along the following lines:

"Second, GCs are thought to operate as a competitive network in which strong feedback inhibition favors a sparse representation that allows only relatively few GCs to be simultaneously active, followed by synaptic modification of the inputs to the GCs that are active as a result of the strong inhibition22,23."

4. Now a more major and more conceptual point. Competitive learning in a neuronal network can take place quite rapidly, and needs only a few presentation of each stimulus that becomes finally represented. Such a process in the brain might occur therefore after a few runs of the treadmill belt. It is therefore a bit of a surprise that changes occur over days, that is rather slowly.

Rather more rapid learning that what is described by the authors would also be beneficial to an episodic memory system, which the hippocampus may provide. That is, even in a novel environment, competitive learning of place filed representations might be advantageous, so that objects represented at the newly coded places could be usefully stored and remembered later. The authors may wish to comment on this.

A suggestion consistent with Rolls' theory of the hippocampus (Rolls 2018; Kesner and Rolls 2015) could be that if the role of the GCs is to help with pattern separation in CA3 for autoassociative learning, then as long as some pattern separation is being performed by the GCs early on, to help select a new set of CA3 cells, then if they continue to improve later that might not matter, because the retrieval cue to the CA3 cells is thought to bypass the DG and reach the CA3 cells by the perforant path (Treves and Rolls, 1992).

Rolls, E. T. (2018) The storage and recall of memories in the hippocampo-cortical system. Cell and Tissue Research 373: 577-604.
Kesner,R.P. and Rolls,E.T. (2015) A computational theory of hippocampal function, and tests of the theory: new developments. Neuroscience and Biobehavioral Reviews 48: 92-147.
Treves,A. and Rolls,E.T. (1992) Computational constraints suggest the need for two distinct input systems to the hippocampal CA3 network. Hippocampus 2: 189-199.


Reviewer #2 (Remarks to the Author):

Kim et al. recorded dentate and CA3 cells while mice repetitively ran on a treadmill for > 20 days. The treadmill was equipped with 6 objects along the length of the belt, and DG cells were classified as granule cells (GCs), mossy cells (MCs), and interneurons. They find that the spatial representations of GC cells were gradually transformed over days to ultimately yield a representation that was homogenous along the length of the track. Subsequent testing on a track with reordered objects and on a track with novel objects then revealed that completely reorganized maps were used for each of these conditions. The fraction of cells with single fields was higher on the reordered than on the novel belt. A relatively simple neuronal network model that includes entorhinal cortex (EC) input to DG, mature GCs, immature GCs, MCs, and interneurons was able to reproduce the patterns of spatial transformation that were observed in experimental data. Overall, the study addresses the long standing question how spatial representations in the dentate gyrus emerge, whether they support pattern separation, and whether there are major differences between cell types in DG. Data acquisition and classification of cell types are performed with high rigor, large populations of cells are recorded from each mouse, and the model proposes a plausible mechanisms on how dentate computations could be performed over the course of many days. In particular, the finding that GC distribution becomes homogenous when inputs are most abundant at landmarks, along with the potential role of MCs to provide the normalizing input is a highly interesting and novel finding. The experimental and computational results can thus be considered a milestone in identifying how DG circuits contribute to intrahippocampal computations. The report is of high quality in its current form, and there are only few details that would warrant additional attention.
(1) Beginning with the second paragraph of the introduction and spanning through the results, the model, the discussion, and supplementary figures, there is an emphasis on grid cells and on landmark vector cells while spatial non-grid cells, which provide the majority of spatial inputs to the hippocampus, are not considered. This omission is particularly glaring when referring to the 3-fold periodicity of periodic cells as reminiscent of grid cells and when assuming that the grid cell input to the hippocampus shows random reorganization of phase. The former detracts from the finding that there are no grid-like cells in any of the hippocampal subregions and that landmark-modulated periodicity could be unrelated to the relatively fixed spacing of true grid cells. The latter seems to make the incorrect assumption that grid phases within a module are reorganized during remapping while not considering the finding that spatial non-grid cells show a substantially higher degree of remapping than grid cells (e.g., Stensola et al., Diehl et al.).
(2) Although previous key publications on DG firing patterns and remapping are cited, there appears to be a missed opportunity in more thoroughly comparing the comprehensive dataset from the present study to the previously published work. In particular, calcium imaging studies (e.g., Hainmueller and Bartos) and electrophysiological recording studies (e.g., Leutgeb et al.) appear to be somewhat conflicting with respect to the degree of remapping in DG and CA3, and it would therefore be valuable to present introduce this topic in more detail and add an in-depth discussion on how the different studies compare.
(3) Although recorded CA3 pyramidal cells are not as numerous as DG cells, it would nonetheless be valuable to report their firing patterns for comparison with the study by Hainmueller et al.
(4) For the recordings on the novel belt, it would be valuable to also analyze the difference between the first and last days.
(5) Discussion: Whether unspecific GC cells correspond to immature GC cells seems quite speculative and the possibility that mature GC may have a range of excitability is not considered.
(6) Discussion: In the final paragraph, the direct input from EC to CA1 and CA3 should also be considered. In some conditions, the CA fields may therefore not depend on DG spatial tuning and context specificity.

Minor points
(1) Legend for Fig. 1b: ‘hilus’ rather than ‘hills’
(2) In the description of the belt (in the paragraph referring to Fig. 2a), it could be explicitly stated that there were 6 landmarks and how they were ordered and spaced. ‘3 pairs’ can be misread as implying that there were 3 landmarks with each landmark consisting of objects mounted on the left and right of the track
(3) Methods: The combination of head fixed recordings with chronic implants seems somewhat unconventional, and perhaps the authors could provide more detail on the rationale for this approach.
(4) Supplementary figure 3a is difficult to decipher. Are the small dots cells in which there was no peak in the crosscorrelation such that these cells could neither be identified as presynaptic or postsynaptic? It also appears that postsynaptic cells were considered to never be excitatory without stating a rationale for this assumption.


Reviewer #3 (Remarks to the Author):

Kim et al combine experimental data from head-fixed animals navigating on a visuo-tactile treadmill with computational modelling in the dentate gyrus. Using classification criteria previously published by the same group, they assign unidentified extracellular recordings to granule cells and mossy cells. Recording sessions performed over consecutive days provide (some) evidence for slow dynamics of spatial representations among the putative GC population, and for an invariant coding of putative mossy cells, whose activity is preferentially locked onto the proximal landmarks. The authors then used a competitive learning model (as in their previous publication) to recapitulate the experimental findings.

1. In my view, the novelty of the present findings is mitigated by previous publications on the topic (Goodsmith et al., 2017; Senzai and Buzsaki., 2017; Danielson et al., 2016 and 2017; Heinmüller and Bartos, 2018). These studies are not properly presented in the present manuscript; therefore, it is not clear to the reader what is the real scientific advance of the present study. The authors should embed their study in the context of previous work, and this should be clearly reflected already in the introduction.
2. The remapping data of the present manuscript are in stark contrast with the results of Senzai and Buzsaki study. In the latter study, the authors used a ‘rate-remapping-like’ configuration, where animals were recorded in different mazes (=changes in local cues) but in the same room (=unchanged distal cues). Since the preparation of Kim et al somewhat mimics the same conditions (i.e. changing proximal cues over belts, unchanged distal cues in the room), one could expect similar findings. However, the results are very different. That is, in the Buzsaki study, GC activity is remarkably stable across environment (also in line with Heinmüller and Bartos, 2018) while mossy cell activity drastically ‘remaps’. This apparent discrepancy should be discussed. Could the difference be simply explained by the different cell classification criteria employed by the two studies?
3. Along the lines of my previous comment: Kim et al used their own classifier for assigning GC and MC identity to unidentified extracellular units. Previous studies, Goodsmith et al 2017 and Senzai and Buzsaki 2017 provided alternative classifiers. This is definitely not helpful and quite confusing from the prospective of the readers, especially if the results of the studies are different. It would be nice to see to which extent these different classification criteria indeed lead to the same classification results - especially considering the fact that the classification criteria of the present study appear to me to be in contrast with previous ones. For example, MCs appear to be more bursty compared to GCs (e.g. Goodsmith paper, Buzsaki paper) while in the present study, quite the opposite seems to be the case. Gamma phase preferences of putative MCs and GCs appear to be also very different between the present study and Senzai and Buzsaki 2017. This should of course not be the case, if both studies are indeed looking at the same cell types. Of course, I don’t expect the authors to solve controversies, since alternative and conflicting results are often important for the advancement of science. However, existing differences should be clearly presented and discussed.
4. In my opinion, an interesting observation of the present study comes from the apparently slow dynamics of the GC representations over consecutive days. However, this finding is in stark contrast with previous work from the Bartos group (Heinmüller and Bartos, 2018). First, by using a similar experimental preparation – also head-fixed animals navigating on a sensory-deprived environment – the Bartos groups finds a quite stable, invariant GC representation over days. There is of course an important methodological difference: in their study, the authors recorded the very same cells across days, which is not the case in the present study (see also next comment below). Second, their remapping data are also quite different from the ones from the present study (Figure 5). These differences should be highlighted and properly discussed (in line with my comment 1 above).
5. The apparent slowness of spatial representation dynamics among the putative GC population is also somewhat surprising – also considering the results from the Bartos group referred to in my comment above. I wonder how much of this apparent ‘slowness’ would actually translate to the ‘real’, freely-moving condition. In the authors’ paradigm (head-fixation + belt + tactile cues), animals are ‘accidentally’ hit by the tactile cues on the treadmill, i.e. the objects just deflect whiskers in a semi-passive manner. Under these conditions, the patterns of exploratory behavior (i.e. object exploration) are of course limited and drastically altered by head-fixation. If the slow dynamics of place field representations are indeed an intrinsic property of the GC network, one should be able to see it also in the natural condition, i.e. freely-moving animals exploring a real environment. In my view, these experiments (if they can be performed by the authors) would indeed be a major addition to the present work, and greatly strengthen the conclusions of the present findings. In my view, it is plausible to conceive that the apparent slowness in the emergence of stable representations in the DG could be somewhat a result of a simplified experimental preparation, and not a reflection of a general computational mechanism of the DG, which operates during natural spatial exploration. If experiments cannot be performed, this point should at least be discussed to in the revised manuscript.
6. The interpretation of the data stemming from consecutive recording sessions (over consecutive days) is not straight-forward to me. Ideally, one would need to monitor the activity of the very same cells across days (as done by Heinmüller and Bartos, 2018) which was of course not possible in the present study (with the exception of Figure 5, i.e. I assume these are the very same cells?). The assumption here is that every day, a statistically representative sample of GC activity is fished out. However, it appears that the units yield changed drastically over days, i.e. few units on the first days of recording, more units later (as evident in figures 3a,b but not commented upon). This limitation should be clearly stated in the revised manuscript. Moreover, and most importantly: is such a relative small number of sampled active cells on the first days sufficient to support the claim about the slow development of GC coding? How is this initial slow sampling coming about: is it the result of technical issues, or do the authors think there are overall less active cells on the first days of exposure to a novel belt? The latter hypothesis would not be supported by their remapping data (Figure 5), where overall the same proportion of cells seems to be active on distinct belts.
7. Along the lines of my previous comment: the limitations of the authors’ preparation should be more thoroughly presented and discussed. The animals are ‘forced’ to pay attention to the proximal cues, since these are the sole cues providing distance information from the reward. Time and space are of course tightly interconnected here, in the presence of stereotypic running speeds and behaviors.
8. The authors tag GCs with the marker POMC (as in their previous study). As the authors acknowledge, this marker is transiently expressed in GCs. Based on the timing of their viral injections relative to the recording time, it appears to me that the authors are preferentially recording from a subpopulation of mature GCs, which are approx. 6 weeks old. Could this bias affect in any way the classification results? This potential limitation should be discussed.
9. Building upon their previous study, the authors recapitulate the present findings in a competitive learning model of the DG network. I find the results interesting, since they point to a clear hypothesis about mossy cell function. However, the model builds upon certain assumptions, which (as such) are not necessarily true. For example, the authors assume that GCs receive grid and object-vector inputs from upstream areas (MEC and LEC, respectively). However, these spatial firing patterns are known to represents only a tiny minority of all firing patterns in the entorinal cortices. Thus, the abundance of grid cells in the model (300 grids onto 3000GC) is disproportionate and not biologically realistic in my opinion. On the other hand, it is clear that the most prominent spatial firing pattern in the superficial layers of MEC and LEC are non-grid spatial neurons, i.e. neurons that are spatially modulated but with irregular spatial firing fields. I would find it more biologically plausible if the authors could model their results assuming spatial non-grid patterns as main inputs, other than the sparse grid and object-vector cells. The transformation the authors are trying to model could be similar to the transformation happening in CA1, where subthreshold spatially-selective inputs covering the entire environment could be selected by plasticity mechanisms (see for example Bittner et al., 2015). The authors should comment on this point.
10. In their model, the authors assume a predominantly inhibitory function of mossy cells on GC activity. This has been long disputed (I am referring here to the ‘dormant basket cells’ and the ‘irritable mossy cells’ hypotheses). The authors should provide stronger evidence for their assumption.

Minor points:
1. Semilunar GCs are mentioned throughout the text, but not elaborated upon. It is my understanding that these cells are quite ‘rare’ and their contribution to MC activity might therefore be minor, if compared with the majority of inputs from the other mature GCs.
2. Prior recordings, animals are trained to run for water rewards on the belt. Neural activity is not recorded during this pre-experimental condition. One could hypothesize that in this condition, an initial GC representation might form (instructed by the distal cues) onto the cue-less belt. This initial formation of place fields (assuming there is one) could be fast. Once the experiment starts and tactile cues are presented on the belt, the GC representation might undergo slower changes to encode the additional local details and features of the environment. Hence, the slow dynamics of spatial coding the authors describe in the present paper. Could the authors comment on this point?
3. Lines 140ff: these classification criteria are not presented here for the first time, but a recapitulation of the authors’ pervious study. I would suggest to shorten it and/or clearly refer to the original source.
4. The authors speculate that cells exhibiting multiple firing fields might correspond to young GCs. This would indeed be in line with the work from the Losonczi group (Danielson et al papers). However, given their distinctive electrophysiological properties, I doubt that mature and young GCs would end up in the same classification cluster.
5. Line 173: cells with low firing have not been included. How low? Could spatial maps be computed and statistical significance of spatial modulation assessed? These data could still be interesting, considering the sparseness of the DG code. Even single spikes could be meaningful (and worth reporting) here, given the strength of downstream detonator synapses.
6. Line 179: tactile cues shrink tubes, Velcro pieces or glues spines. It would be great to have pictures of these cues shown in the suppl materials. It would facilitate reproduction of the experimental procedures and experimental data by other groups (catalogue numbers could also be provided)
7. Figure 2c: I would suggest to present the panels in the same order they are referred to in the text (lines 183ff) or vice versa
8. Lines 206: n=4. What does it refer to? N=4 animals or sessions or animals?
9. Line 268: ‘field extension is preceded by a gradual reduction in the in-field rate’ I do not really see it in the corresponding figure 4c. Both transitions appear equally sharp to me. Could maybe the authors quantify that?
10. Line 283: ‘new set of landmarks’. Please specify, ideally with pictures (see comment 4 above)
11. Line 487: Hebbian synaptic plasticity and postsynaptic firing are referred to in the context of place field emergence. This could be embedded into the Bittner et al NatNeursc 2015; Diamantaki et al CurrBiol 2016; and Monaco et al., NatNeurosc 2014. Postsynaptic firing of principal cells (mediated by dishinibition?) could indeed play a crucial role here, also according to the behavioral data from the Knierim paper (i.e. potentiation of place fields during novelty-induced head-scanning behavior; Monaco et al., 2014).
12. Lines 512ff: the authors speculate that young GCs could receive inputs from mature GCs. Do the authors have evidence for monosynaptic excitatory connectivity between the putative mature GCs and the putative young GCs (as early speculated, the cells with multiple firing fields, line 303)?

**Author rebuttal, first version:**

Reviewers' comments:

Reviewer #1 (Remarks to the Author):

1. This is an interesting test of the theory of how competitive learning in DG sets up place cell activity from grid cell activity in EC.

The following comments are provided as feedback to help the authors improve the paper.

We thanks the reviewer for the helpful suggestions to improve the manuscript.

2. Line 73: "How such activity patterns emerge in the DG, the first processing stage of the hippocampal trisynaptic loop, is a fundamental question largely unresolved."

This could be stated more correctly as follows:

"How such activity patterns emerge in the DG, the first processing stage of the hippocampal trisynaptic loop, is a fundamental question. There is a clear theory that competitive learning in the DG and also at other stages of the hippocampus implements the transform (Rolls et al 2006; Si and Treves 2009), but empirical tests of the theory are still needed).

We agree with the reviewer. The introduction has been considerably modified to address all reviewers’ comments and the above suggestion was incorporated.

3. "Second, GCs are thought to operate as a competitive network in which strong feedback inhibition favors a ‘winner-take-all’ process and allows only a few GCs to be simultaneously active22,23."

The "winner-take-all' statement is not how the theory is held to apply to the brain, as it is most unlikely that neuronal systems ever produce a single winner, and that is not part of the theory. Nor does competitive learning involved only inhibition: it also involves synaptic modification, learning. I expect that the authors are fully aware of this, and the statement would be greatly improved by wording along the following lines:

"Second, GCs are thought to operate as a competitive network in which strong feedback inhibition favors a sparse representation that allows only relatively few GCs to be simultaneously active, followed by synaptic modification of the inputs to the GCs that are active as a result of the strong inhibition22,23."

In the revised introduction, we do not use the statement “winner take all” and we emphasize that the combination of cell competition and Hebbian synaptic modification is critical.

4. Now a more major and more conceptual point. Competitive learning in a neuronal network can take place quite rapidly, and needs only a few presentation of each stimulus that becomes finally represented. Such a process in the brain might occur therefore after a few runs of the treadmill belt. It is therefore a bit of a surprise that changes occur over days, that is rather slowly.

Rather more rapid learning that what is described by the authors would also be beneficial to an episodic memory system, which the hippocampus may provide. That is, even in a novel environment, competitive learning of place filed representations might be advantageous, so that objects represented at the newly coded places could be usefully stored and remembered later. The authors may wish to comment on this.

A suggestion consistent with Rolls' theory of the hippocampus (Rolls 2018; Kesner and Rolls 2015) could be that if the role of the GCs is to help with pattern separation in CA3 for autoassociative learning, then as long as some pattern separation is being performed by the GCs early on, to help select a new set of CA3 cells, then if they continue to improve later that might not matter, because the retrieval cue to the CA3 cells is thought to bypass the DG and reach the CA3 cells by the perforant path (Treves and Rolls, 1992).

Rolls, E. T. (2018) The storage and recall of memories in the hippocampo-cortical system. Cell and Tissue Research 373: 577-604.

Kesner,R.P. and Rolls,E.T. (2015) A computational theory of hippocampal function, and tests of the theory: new developments. Neuroscience and Biobehavioral Reviews 48: 92-147.

Treves,A. and Rolls,E.T. (1992) Computational constraints suggest the need for two distinct input systems to the hippocampal CA3 network. Hippocampus 2: 189-199.

We thanks the reviewer for raising this interesting point. We now address these issues in the discussion (paragraph 5), following some of the reviewer’s suggestions.

Regarding how object location in novel environments might be stored:

While the time for the GC development to reach the plateau is quite long, it doesn’t prevent that some learning still happen early on before the plateauing, such that information might already be stored after a few trials and used to retrieve object locations in relatively novel environments. The long time period to plateau only suggests that memory for object layouts improves with time and become optimal after ~10 days, under these experimental conditions.

Another possibility is that faster learning largely occur in the CA regions while the contribution of DG to fast learning is mostly through DG pattern separation assisting the CA3 region to store information in a new set of CA3 cells.

The theory of Treves and Rolls 1992 stipulating that the retrieval cue to the CA3 cells might bypass the DG and reach the CA3 cells by the perforant path would indeed help explain how the recall of previously stored information is not impaired by the slow changes in the DG. We would prefer not to elaborate on this issue since we are not sure of several factors: 1) this problem seems pertinent specifically for the encoding of transient information, as for stable cues, the slow persistent changes should improve their encoding and memory recall; 2) we are not sure if/how transient information is stored in the DG; and 3) we are not sure how much the slow learning really affect previous information storage.

We would like to thank the reviewer for his helpful comments.

Reviewer #2 (Remarks to the Author):

Kim et al. recorded dentate and CA3 cells while mice repetitively ran on a treadmill for > 20 days. The treadmill was equipped with 6 objects along the length of the belt, and DG cells were classified as granule cells (GCs), mossy cells (MCs), and interneurons. They find that the spatial representations of GC cells were gradually transformed over days to ultimately yield a representation that was homogenous along the length of the track. Subsequent testing on a track with reordered objects and on a track with novel objects then revealed that completely reorganized maps were used for each of these conditions. The fraction of cells with single fields was higher on the reordered than on the novel belt. A relatively simple neuronal network model that includes entorhinal cortex (EC) input to DG, mature GCs, immature GCs, MCs, and interneurons was able to reproduce the patterns of spatial transformation that were observed in experimental data. Overall, the study addresses the long standing question how spatial representations in the dentate gyrus emerge, whether they support pattern separation, and whether there are major differences between cell types in DG. Data acquisition and classification of cell types are performed with high rigor, large populations of cells are recorded from each mouse, and the model proposes a plausible mechanisms on how dentate computations could be performed over the course of many days. In particular, the finding that GC distribution becomes homogenous when inputs are most abundant at landmarks, along with the potential role of MCs to provide the normalizing input is a highly interesting and novel finding. The experimental and computational results can thus be considered a milestone in identifying how DG circuits contribute to intrahippocampal computations. The report is of high quality in its current form, and there are only few details that would warrant additional attention.

(1) Beginning with the second paragraph of the introduction and spanning through the results, the model, the discussion, and supplementary figures, there is an emphasis on grid cells and on landmark vector cells while spatial non-grid cells, which provide the majority of spatial inputs to the hippocampus, are not considered.

We thanks the reviewer for raising these points and bringing attention to the spatial non-grid cells (which we missed).

To address the reviewer comment, we have implement a new version of the model that incorporate the spatial non-grid cells in EC (Figure 8). This cell population is actually critical to reproduce the emergence of the ‘unspecific’ place field cells (cells with multiple fields that are not LV or periodic). Since it was not possible to reproduce the initial preponderance of LV and periodic patterns in GCs when spatial non-grid cells were active from the beginning, we had to assume a progressive development for this group of cells. Such development is plausible considering local learning in EC and the buildup of spatial representations in the hippocampus that feedback to the EC.

This omission is particularly glaring when referring to the 3-fold periodicity of periodic cells as reminiscent of grid cells and when assuming that the grid cell input to the hippocampus shows random reorganization of phase. The former detracts from the finding that there are no grid-like cells in any of the hippocampal subregions and that landmark-modulated periodicity could be unrelated to the relatively fixed spacing of true grid cells.

We agree that the link between periodic cells and grid cells is not certain.

A similarity of periodic cells to grid cell activity patterns in 1-dimensional environments is assessable in the paper of Derdikman et al. 2009 (Nat Neurosci 12, 1325-1332, Fig. 1 and 2), where some grid cells identified in a 2-dimensional arena show periodic activity within the linear alleys of a ‘hair pin’ maze. We now refer to that study when pointing out the similarity of periodic cells with grid cells.

While grid patterns are not typically observed in the hippocampus, it should be noted that our experimental conditions are different than typical place cell recording conditions. 1) We used a treadmill in which the spatial information is probably oversimplified, resulting in less convergence of diverse information and a better chance to detect individual spatial mechanisms. 2) We recorded cell activity from the very beginning of the learning of the belt whereas place cell recordings are typically performed in familiar environments. Periodic cells in the treadmill almost completely disappeared after the first day, consistent with the fact that they are not seen in the typically familiar environments.

These points are now incorporated in the discussion (paragraph 6).

The latter seems to make the incorrect assumption that grid phases within a module are reorganized during remapping while not considering the finding that spatial non-grid cells show a substantially higher degree of remapping than grid cells (e.g., Stensola et al., Diehl et al.).

We have corrected the model according to the reviewer’s comment. While grid phases are not anymore reorganized across the different belt layouts, the spatial non-grid cells are remapping (Figure 8).

(2) Although previous key publications on DG firing patterns and remapping are cited, there appears to be a missed opportunity in more thoroughly comparing the comprehensive dataset from the present study to the previously published work. In particular, calcium imaging studies (e.g., Hainmueller and Bartos) and electrophysiological recording studies (e.g., Leutgeb et al.) appear to be somewhat conflicting with respect to the degree of remapping in DG and CA3, and it would therefore be valuable to present introduce this topic in more detail and add an in-depth discussion on how the different studies compare.

As suggested by the reviewer, we outline in the discussion (paragraphs 2-3) how our results compare to that of previous studies.

Briefly,

In terms of number of place fields, our findings are consistent with recent studies suggesting that MCs display several firing fields while GCs have less, typically single, place fields. Of the GCs having spatially modulated activity, the fraction showing single firing fields in our study (64% on days 10-20) was similar to the ones in the studies of Senzai & Buzsaki 2017 (~66%) and GoodSmith et al. 2017 (~70%), but higher to the one reported by Leutgeb et al. 2007 (39%). It is possible that the lower proportion of single-field GCs in the study of Leutgeb et al. 2007 is because some of the GCs were actually MCs. Another reason might be a difference in the familiarity of the environment, as the morphing experiments carried in the study of Leutgeb et al. 2007 might introduce a novelty component that reduce the proportion of single-field cells, considering our observation that single-field cells decreased in the new belt layouts.

In terms of temporal dynamic, only the study of Hainmuller & Bartos 2018 monitored GC activity across multiple days. In that study, recordings in a first environment started after >10 training days such that the effect of learning is assessable only when the animals are introduced in a second ‘novel’ environment. While the analyses were designed mostly to assess differences in stability between hippocampal regions, it is noteworthy that the slow increase in spatial correlation for pairs of consecutive days (Hainmuller & Bartos 2018; figure 4b) is consistent with the progressive development we observe: the initially high rate of change we observe match the initially low spatial correlations, while the plateauing we observe match the increased spatial correlation.

The remapping properties of GCs are consistent with other studies. In particular, the observation that cells with single fields tended to represent a single environment while cells with multiple fields encoded several environments is consistent with GoodSmith et al. 2017, and the overall drop in the number of spatially modulated cells in the novel environments is also observed in the study of Hainmuller & Bartos 2018 (figure 1f). It is noticeable that the extent of DG remapping, when compared to CA3, differs depending on the study. The studies of Senzai & Buzsaki 2017 and Hainmuller & Bartos 2018 report more remapping in CA3 while Leutgeb et al. 2007 report more remapping in DG. This discrepancy might result from the inclusion of some MCs in the GC population in Leutgeb et al. 2007, but also from the distinct ways the environments were altered: environmental cues (mostly local ones) were altered in Senzai & Buzsaki 2017 and Hainmuller & Bartos 2018, while the shape of the maze boundaries was modified in Leutgeb et al. 2007.

(3) Although recorded CA3 pyramidal cells are not as numerous as DG cells, it would nonetheless be valuable to report their firing patterns for comparison with the study by Hainmueller et al.

As suggested by the reviewer, we have included a new figure (supplementary Figure 5) and a result section for the putative CA3 cells (See the result section ‘Representations of MCs and CA3 cells’).

(4) For the recordings on the novel belt, it would be valuable to also analyze the difference between the first and last days.

As suggested by the reviewer, we have modified the figure 5 panels b-d to show the information for 2 time periods.

It should be mentioned that no clear trend across sessions was visible when the analyses were carried on individual sessions, due to large session variability (which could be explained by an overall decrease in detected cells >20 days after the silicon probe implantation). Therefore we pulled data from the first and last 3 days in the novel belts.

Since little differences were visible between the 2 time periods and since it was not clear if these differences were due to session variability, the small number of recording days in the new belts, or other reasons, we preferred not to elaborate on differences between first and last days and focus only on the differences across belts.

(5) Discussion: Whether unspecific GC cells correspond to immature GC cells seems quite speculative and the possibility that mature GC may have a range of excitability is not considered.

We agree with the reviewer. We now refer to a subgroup of GCs with higher excitability, which might correspond to immature GCs and/or simply mature GCs with higher excitability. The new model in Figure 8, which incorporate the non-grid spatial cells in EC, is compatible with either of these scenarios, as it does not include mature-to-young GC connections.

(6) Discussion: In the final paragraph, the direct input from EC to CA1 and CA3 should also be considered. In some conditions, the CA fields may therefore not depend on DG spatial tuning and context specificity.

We agree with the reviewer that direct inputs from EC might be sufficient to support the spatial representations in the CA1-CA3 regions. We now simply mention that cells in the CA regions might depend on DG output for spatial tuning and context specificity of firing fields ‘under certain conditions’.

Minor points

(1) Legend for Fig. 1b: ‘hilus’ rather than ‘hills’

We thank the reviewer. This was corrected.

(2) In the description of the belt (in the paragraph referring to Fig. 2a), it could be explicitly stated that there were 6 landmarks and how they were ordered and spaced. ‘3 pairs’ can be misread as implying that there were 3 landmarks with each landmark consisting of objects mounted on the left and right of the track

We have modified the text to avoid this confusion. Also, we have incorporated a new figure (Supplementary Figure 4) with pictures and schemes of the treadmill, landmarks and belt layouts.

(3) Methods: The combination of head fixed recordings with chronic implants seems somewhat unconventional, and perhaps the authors could provide more detail on the rationale for this approach.

As suggested by the reviewer, we now provide more details on the rational of the treadmill, both in the introduction (paragraph 3) and methods (‘Treadmill apparatus and behavioral paradigm’).

The treadmill presents several advantage for our study:

Compared to freely moving conditions, the animal behavior and trajectory across landmarks are very consistent over trials and sessions. This should allow a better assessment of learning-induced changes.

The spatial information is simpler: the only environmental cues that can be used to implement position on the belt are the landmarks that are fixed on the belt, and the mice experience the landmarks one at a time. As a result, spatial mechanisms (LV, periodic, unspecific, single) are clearly separable, a clear transformation of spatial representations is detectable, and the interpretation of data using models is simpler.

While the treadmill is less natural than freely-moving conditions, running on the treadmill belt might be comparable to mice running (a circular trajectory) in high grass or underground tunnels where local cues and path-integration are mostly available.

(4) Supplementary figure 3a is difficult to decipher. Are the small dots cells in which there was no peak in the crosscorrelation such that these cells could neither be identified as presynaptic or postsynaptic?

The small dots represent all the cells recorded. Among those, some were identified as excitatory and excited based on the peak in the crosscorrelation. The symbols for all cells (small grey dots), excitatory cells (large green dots) and excited cells (purple circles) were simply overlaid.

We have added the label ‘all cells’ for the small gray dots in the figure panel, beside excitatory cells (large green dots) and excited cells (purple circles), to improve the clarity.

It also appears that postsynaptic cells were considered to never be excitatory without stating a rationale for this assumption.

The same cell could actually be both excitatory and excited. This is visible by the superposition of a green dot and a purple circle for some of the cells.

We thank the reviewer for the constructive comments.

Reviewer #3 (Remarks to the Author):

Kim et al combine experimental data from head-fixed animals navigating on a visuo-tactile treadmill with computational modelling in the dentate gyrus. Using classification criteria previously published by the same group, they assign unidentified extracellular recordings to granule cells and mossy cells. Recording sessions performed over consecutive days provide (some) evidence for slow dynamics of spatial representations among the putative GC population, and for an invariant coding of putative mossy cells, whose activity is preferentially locked onto the proximal landmarks. The authors then used a competitive learning model (as in their previous publication) to recapitulate the experimental findings.

1. In my view, the novelty of the present findings is mitigated by previous publications on the topic (Goodsmith et al., 2017; Senzai and Buzsaki., 2017; Danielson et al., 2016 and 2017; Heinmüller and Bartos, 2018). These studies are not properly presented in the present manuscript; therefore, it is not clear to the reader what is the real scientific advance of the present study. The authors should embed their study in the context of previous work, and this should be clearly reflected already in the introduction.

We agree with the reviewer that our study overlaps in some respects with previous studies that monitored the spatial activity of GCs and MCs. We now recapitulate the findings from previous studies in the introduction (paragraph 1) and point out the differences and consistencies of our results with previous studies in the discussion (paragraph 2 and 3).

We also formulate more clearly in the introduction (paragraph 1) and discussion (paragraph 1 and 3) the novel aspects of our study. The novelty of our study relative to these previous studies is that we investigate the development of the dentate spatial map during the learning of an environment (using both long-term recording and modeling approaches). Only the study of Heinmüller and Bartos 2018 previously monitored GC activity across multiple days, however, the analyses were mainly designed to compare the stability of spatial representations between hippocampal regions.

2. The remapping data of the present manuscript are in stark contrast with the results of Senzai and Buzsaki study. In the latter study, the authors used a ‘rate-remapping-like’ configuration, where animals were recorded in different mazes (=changes in local cues) but in the same room (=unchanged distal cues). Since the preparation of Kim et al somewhat mimics the same conditions (i.e. changing proximal cues over belts, unchanged distal cues in the room), one could expect similar findings. However, the results are very different. That is, in the Buzsaki study, GC activity is remarkably stable across environment (also in line with Heinmüller and Bartos, 2018) while mossy cell activity drastically ‘remaps’. This apparent discrepancy should be discussed. Could the difference be simply explained by the different cell classification criteria employed by the two studies?

We think that because we did not described the treadmill apparatus properly, understanding the spatial mechanisms in the treadmill was not straightforward. We now provide more details on the rational and mechanisms of the treadmill in the introduction (paragraph 3) and methods (‘Treadmill apparatus and behavioral paradigm’).

The treadmill environment is actually quite different than an open maze, and changing the layout of cues on the treadmill belt is expected to have a much larger impact than changing local cues in an open maze (as done in the study of Senzai and Buzsaki 2017). Indeed, following change in local cues in an open maze, distal room cues and maze boundaries can still supply spatial information to estimate position in the maze, therefore the GCs might be able to maintain their place fields. In the treadmill, only the cues fixed on the belt (i.e. moving together with the belt) can be used for mapping positions on the belt. Therefore altering the layout of cues on the belt should have a large impact on place fields. This point is addressed in the paragraph 3 of the discussion.

The experimental conditions in the study of Heinmüller and Bartos 2018 are likely more similar to the treadmill conditions. Although they emphasizes smaller remappings for GCs than for CA1/CA3 cells, they nevertheless observe a substantial remapping of GCs (in their figure 2d, F-N versus F-F). Also, similar to our findings, they report a decrease in the number of GC place cells for the novel environment relative to the familiar environment (figure 1f in their paper; figure 5b in the current manuscript).

Please see below regarding difference in classification criteria.

3. Along the lines of my previous comment: Kim et al used their own classifier for assigning GC and MC identity to unidentified extracellular units. Previous studies, Goodsmith et al 2017 and Senzai and Buzsaki 2017 provided alternative classifiers. This is definitely not helpful and quite confusing from the prospective of the readers, especially if the results of the studies are different. It would be nice to see to which extent these different classification criteria indeed lead to the same classification results - especially considering the fact that the classification criteria of the present study appear to me to be in contrast with previous ones. For example, MCs appear to be more bursty compared to GCs (e.g. Goodsmith paper, Buzsaki paper) while in the present study, quite the opposite seems to be the case. Gamma phase preferences of putative MCs and GCs appear to be also very different between the present study and Senzai and Buzsaki 2017. This should of course not be the case, if both studies are indeed looking at the same cell types. Of course, I don’t expect the authors to solve controversies, since alternative and conflicting results are often important for the advancement of science. However, existing differences should be clearly presented and discussed.

We now discuss the discrepancies and similarities of our results with previous studies (paragraph 2 and 3 of the discussion). It is possible that the differences point out by the reviewer reflect discrepancies in cell classification. However, it is not necessarily the case given some unknown factors that we point out below.

Regarding burst activity:

First, it is important to mention that the profiles of MC autocorrelograms in our study match quite well the one reported in Henze and Buzsaki 2007, where MCs were recorded and labeled using intracellular electrodes. This could be appreciated on a simple visual display of all the autocorrelograms of DRD2-light excited cells (Jung et al 2019 Sci Rep 9:9545).

Goodsmith et al., 2017 compute the burst indexes from the histogram of inter-spike intervals, using the definition ‘burst index = the fraction of ISI <= 6ms’ and report in Figure S4 higher burst indexes in the hilus than in the GCL, which seems in disagreement with our Figure 1d. There are several factors that need to be considered:

1) The method Goodsmith et al., 2017 used for calculating burst indexes might not only reflect the burst activity but also the refractory periods of the cells. Some highly bursting cells may have refractory periods > 6ms and show low burst indexes with this method.
2) Burst indexes are shown for ‘hilus’ and ‘GCL’ cells, which is not clearly equivalent to ‘MCs’ and ‘GCs’.
3) Most importantly, their recordings include both periods of sleep and maze exploration, while our recordings are during wake/treadmill running and probably include more frequent passage through place fields’ peak position. These behavioral factors might impact on cell bursting activity and refractory periods.

Senzai and Buzsaki 2017 estimate a burst index based on the spike autocorrelogram. According to the description in their Figure 1A legend, the burst index is estimated as a/b, with a = the sum of the 3,4,5ms bins, and b = the mean of the 200 to 300ms bins. They do not compare the burst indexes of MCs and GCs in the manuscript, however, the burst indexes chosen as criteria for both GCs and MCs seem relatively high.

1) As for Goodsmith et al., 2017, this method might not only reflect the burst activity but also the refractory periods of the cells. Also, given that the factors a and b do not have equivalent dimensions (a sum of 3 bins versus an average of 100 bins), some caution is required regarding how the burst indexes reflect the magnitude of burst activity.
2) As for Goodsmith et al., 2017, their recordings include both periods of sleep and maze exploration.

Regarding gamma oscillations:

To some degree, our results are consistent with Senzai and Buzsaki 2017: in both studies, MCs and GCs showed different gamma phase preference, and GCs were more modulated by gamma oscillations and coupled with gamma power than MCs. However, we agree that the difference in gamma phase preference is larger in our study. This could reflect some cell classification differences but also difference in the location gamma oscillations were measured. We were careful to choose recording sites located deep in the hilus since they optimally segregated GCs and MCs. Difference in behavioral state might also contribute, as their recordings included sleep epochs.

It is noteworthy that other aspects are quite consistent between the 3 studies despite the different classification methods. Consistent with these two studies, MCs displayed several firing fields while GCs displayed mostly a single place field on days 10-20 (once the environment was familiar). The ratio of GCs showing single versus multiple firing fields in our study (64% on days 10-20) was similar to that in the studies of Senzai & Buzsaki 2017 (~66%) and GoodSmith et al. 2017 (~70%).

4. In my opinion, an interesting observation of the present study comes from the apparently slow dynamics of the GC representations over consecutive days. However, this finding is in stark contrast with previous work from the Bartos group (Heinmüller and Bartos, 2018). First, by using a similar experimental preparation – also head-fixed animals navigating on a sensory-deprived environment – the Bartos groups finds a quite stable, invariant GC representation over days. There is of course an important methodological difference: in their study, the authors recorded the very same cells across days, which is not the case in the present study (see also next comment below). Second, their remapping data are also quite different from the ones from the present study (Figure 5). These differences should be highlighted and properly discussed (in line with my comment 1 above).

This is indeed an important point to stress, which we now address in the discussion (paragraph 3). As we explain below, our results are actually consistent with the data of Heinmuller and Bartos 2018, even though they might seem different at first glance.

It should be note that a perfect comparison between the two studies is not straightforward since their analyses were mainly designed to compare the stability of the spatial representations between hippocampal regions and since we did not perform spatial correlation analyses over consecutive days (as they did) because cell identity couldn’t be tracked over days.

Regarding the slow dynamics:

First we should point out that the recordings started after >10 days of training in the first environment of Heinmuller and Bartos 2018. We recorded from day 1 and the development of the spatial map reached a plateau at ~day 10. Hence the stability of GC representation emphasized for the first environment in Heinmuller and Bartos 2018 is not inconsistent with our results.

The animals were then introduced to a novel environment in Heinmuller and Bartos 2018. At that stage, the conditions might resemble the ones in our study, but are still not exactly similar since novel and familiar environments seemed to have a lot of shared features. Some learning from the familiar environment might have been transferred to the novel environment, like we found in our study. Nevertheless, some slow dynamics are visible in their data (notice the slow increase in spatial correlation in their figure 4b). The spatial correlations were measured between consecutive days and were initially low (consistent with the high rate of change we observed on the first days) and then increased (consistent with the lower rate of change as the spatial representation is approaching the plateau).

Regarding the remappings:

As mentioned earlier, the remapping data of the 2 studies are quite consistent. Heinmuller and Bartos 2018 observe a substantial remapping of GCs (in their figure 2d, F-N versus F-F). Also, similar to our findings, they report a decrease in the number of GC place cells for the novel environment relative to familiar environment (figure 1f in their paper; figure 5b in the current manuscript).

5. The apparent slowness of spatial representation dynamics among the putative GC population is also somewhat surprising – also considering the results from the Bartos group referred to in my comment above. I wonder how much of this apparent ‘slowness’ would actually translate to the ‘real’, freely-moving condition. In the authors’ paradigm (head-fixation + belt + tactile cues), animals are ‘accidentally’ hit by the tactile cues on the treadmill, i.e. the objects just deflect whiskers in a semi-passive manner. Under these conditions, the patterns of exploratory behavior (i.e. object exploration) are of course limited and drastically altered by head-fixation. If the slow dynamics of place field representations are indeed an intrinsic property of the GC network, one should be able to see it also in the natural condition, i.e. freely-moving animals exploring a real environment. In my view, these experiments (if they can be performed by the authors) would indeed be a

major addition to the present work, and greatly strengthen the conclusions of the present findings. In my view, it is plausible to conceive that the apparent slowness in the emergence of stable representations in the DG could be somewhat a result of a simplified experimental preparation, and not a reflection of a general computational mechanism of the DG, which operates during natural spatial exploration. If experiments cannot be performed, this point should at least be discussed to in the revised manuscript.

We now addressed the slow dynamics in the discussion (paragraph 5).

It is important to mention that the treadmill is not motorized, that the mice move themselves the treadmill belt and that the belt stretches 30-cm ahead of the mice position so that the mice can see each cue approaching, from 30-cm ahead before it touches their whiskers. Therefore the experience of the cues is not totally passive.

It is indeed possible that the dynamics are slower in the treadmill compared to real environments, because of the paucity of cues and the restriction of head-scanning movements. But it is also likely that the dynamics differ among real environments as well, depending on the availability of cues and attention state. These are important questions that need to be explored in the future, however we feel that such investigation would be out of the scope of the current study and might take years to conduct, considering the poor reliability of mice behavior in freely moving conditions and the difficulty to carry silicon probe recordings in freely moving mice over week-long periods.

The slow dynamic is actually not inconceivable considering the amount of time/visits required to know perfectly an environment. For instance, a human equivalent to the observed learning of the treadmill layout might be the familiarization to the sequence of stores and restaurants along a long street (that is narrow and zigzagging such that mostly local cues are visible). Such learning might take weeks to become optimal, if no special effort is made to memorize the places.

6. The interpretation of the data stemming from consecutive recording sessions (over consecutive days) is not straight-forward to me. Ideally, one would need to monitor the activity of the very same cells across days (as done by Heinmüller and Bartos, 2018) which was of course not possible in the present study

We agree that recording the same cell population across multiple days would be optimal as it would allow to follow the evolution of individual cells. Still, we could analyze the evolution of individual cells across trials within the sessions (Figure 4) and the results are consistent with the evolution observed at the population level.

(with the exception of Figure 5, i.e. I assume these are the very same cells?).

These are the same cells.

The assumption here is that every day, a statistically representative sample of GC activity is fished out. However, it appears that the units yield changed drastically over days, i.e. few units on the first days of recording, more units later (as evident in figures 3a,b but not commented upon). This limitation should be clearly stated in the revised manuscript. Moreover, and most importantly: is such a relative small number of sampled active cells on the first days sufficient to support the claim about the slow development of GC coding? How is this initial slow sampling coming about: is it the result of technical issues, or do the authors think there are overall less active cells on the first days of exposure to a novel belt? The latter hypothesis would not be supported by their remapping data (Figure 5), where overall the same proportion of cells seems to be active on distinct belts.

In the figure 3a,b, we show only the cells that exhibited place fields. The number of cells detected (i.e. active cells) is much larger and doesn’t increase over days. The purpose of showing only cells with place fields in these color-coded maps is for the reader to better see the firing field patterns (the line representing the rate map of one cell would be too thin if all cells were included in these color-coded plots). The fraction of GCs exhibiting place fields, relative to all detected GCs in each session, is shown in figure 3c.

We apologize that the figure legend and previous label ‘cells’ for the y axis of Figure 3a were confusing. We have improved the figure legend and labeling of the y axis, specifying more clearly that these cells are GCs exhibiting place fields.

7. Along the lines of my previous comment: the limitations of the authors’ preparation should be more thoroughly presented and discussed. The animals are ‘forced’ to pay attention to the proximal cues, since these are the sole cues providing distance information from the reward. Time and space are of course tightly interconnected here, in the presence of stereotypic running speeds and behaviors.

As suggested by the reviewer, we have added some details and rational regarding the treadmill in the introduction and methods, as well as a new figure (Supplementary figure 4).

As the reviewer point out, the cues fixed on the belt and the mechanism of path-integration are providing the only spatial information that can be used to implement position on the belt. Such situation might not be too different for mice than running within narrow channels underground or in high grass, where distal cues are not available.

8. The authors tag GCs with the marker POMC (as in their previous study). As the authors acknowledge, this marker is transiently expressed in GCs. Based on the timing of their viral injections relative to the recording time, it appears to me that the authors are preferentially recording from a subpopulation of mature GCs, which are approx. 6 weeks old. Could this bias affect in any way the classification results? This potential limitation should be discussed.

We now discuss this possibility (paragraph 2 of the discussion).

It is indeed possible that ‘younger’ GCs were targeted by our POMC-Cre dependent expression. To what extent these cells became completely mature at the time of the recordings is not totally clear, given that the POMC gene is believed to be expressed during the first month after cell mitosis and that the recordings were performed 4 weeks after the virus injection.

Our cell classification is not only based on the fact that the dense cluster of putative GCs was matched by POMC-light excited cells, but also by the fact that it was largely underrepresented by the DRD2 light-excited cells and that putative GCs were located more superficially relative to putative MCs.

9. Building upon their previous study, the authors recapitulate the present findings in a competitive learning model of the DG network. I find the results interesting, since they point to a clear hypothesis about mossy cell function. However, the model builds upon certain assumptions, which (as such) are not necessarily true. For example, the authors assume that GCs receive grid and object-vector inputs from upstream areas (MEC and LEC, respectively). However, these spatial firing patterns are known to represents only a tiny minority of all firing patterns in the entorinal cortices. Thus, the abundance of grid cells in the model (300 grids onto 3000GC) is disproportionate and not biologically realistic in my opinion. On the other hand, it is clear that the most prominent spatial firing pattern in the superficial layers of MEC and LEC are non-grid spatial neurons, i.e. neurons that are spatially modulated but with irregular spatial firing fields. I would find it more biologically

plausible if the authors could model their results assuming spatial non-grid patterns as main inputs, other than the sparse grid and object-vector cells. The transformation the authors are trying to model could be similar to the transformation happening in CA1, where subthreshold spatially-selective inputs covering the entire environment could be selected by plasticity mechanisms (see for example Bittner et al., 2015). The authors should comment on this point.

We thanks the reviewer for raising this point.

To address the reviewer comment, we have implemented a new model in which the spatial non-grid cells of the EC are included (Figure 8). The proportions of the different cell types relatively match the proportions reported.

The spatial non-grid cells allowed to reproduce the emergence of the ‘unspecific’ place field cells (cells with multiple fields that are not LV or periodic). However, it was not possible to reproduce the initial preponderance of LV and periodic patterns in GCs when the spatial non-grid cells were active from the beginning. Therefore we had to assume a progressive development for this group of cells. Such development is plausible considering local learning in EC and the buildup of spatial representations in the hippocampus that feedback to the EC.

10. In their model, the authors assume a predominantly inhibitory function of mossy cells on GC activity. This has been long disputed (I am referring here to the ‘dormant basket cells’ and the ‘irritable mossy cells’ hypotheses). The authors should provide stronger evidence for their assumption.

We thank the reviewer for raising this point. We agree that the net excitatory versus inhibitory effect of the MCs on the GCs has been disputed and that some caution is required. However, to the best of our knowledge, most recent studies using rapid genetic degeneration of mossy cells or optogenetic stimulation of the MCs supported a predominant inhibitory effect of MCs (Jinde et al. 2012, Neuron 76:1189-1200; Hsu et al. 2016, Cerebral Cortex 26: 2715-2727; Bui et al. 2018, Science 359:787-790). Notably, the Bui et al 2018 study comes from the same lab that previously produced strong evidence for a predominant excitatory effect of MCs (Ratzliff et al. 2004, J Neurosci 24:2259-2269). In terms of the scale of the inhibitory predominance, the Hsu et al. 2016 study, that measured both excitatory and inhibitory currents in GCs generated by the optogenetic stimulations of MCs or EC cells, found a I/E ratio of 4.2 for MC stimulations while a I/E ratio of 0.6 for EC stimulations.

We now refer to these studies and raise some caution when assuming the predominant inhibitory effect of MCs.

Minor points:

1. Semilunar GCs are mentioned throughout the text, but not elaborated upon. It is my understanding that these cells are quite ‘rare’ and their contribution to MC activity might therefore be minor, if compared with the majority of inputs from the other mature GCs.

As suggested by the reviewer, we provide more information for semilunar GCs in the discussion (paragraph 3).

A study from Larimer and Strowbridge 2010 (Nat Neurosci 13, 213-222) has reported that the population of seminal GCs is particularly responsive to perforant path stimulations (compared to other GCs) and generates large barrage of excitatory postsynaptic potentials in MCs.

Because the MCs but not GCs overrepresented the cues, remap more than the GC population (Senzai and Buzsaki 2017; Goodsmith et al., 2017; Jung et al. 2019), and do not seem to inherit spatial activity from the GCs that provide excitation (Senzai and Buzsaki 2017), inputs other than GC-to-MC need to be involved to explain the MC activity patterns. Among possible inputs, semilunar GCs, EC cells and CA3 cells are plausible candidates.

2. Prior recordings, animals are trained to run for water rewards on the belt. Neural activity is not recorded during this pre-experimental condition. One could hypothesize that in this condition, an initial GC representation might form (instructed by the distal cues) onto the cue-less belt. This initial formation of place fields (assuming there is one) could be fast. Once the experiment starts and tactile cues are presented on the belt, the GC representation might undergo slower changes to encode the additional local details and features of the environment. Hence, the slow dynamics of spatial coding the authors describe in the present paper. Could the authors comment on this point?

As we mentioned earlier, it is hard to conceive how the room cues, even if visible (black panels flanked both sides of the belt), could provide spatial information useful for mapping positions on the treadmill belt. The mice are head-fixed such that they remain static relative to the room cues as they run. Hence place cells might only encode position on the belt via two mechanisms: 1) the sensing of the cues fixed on the belt and 2) the estimation of traveled distance via self-motion information (path-integration).

It is conceivable that a spatial map entirely based on self-motion information could emerge in the cue-less belt when the animals are trained to be head-fixed on the belt and to run for water rewards. However, based on previous studies and our own observations, very few spatially modulated cells are detected in cue-less belts and these cells encoded periodic intervals (Villette et al. 2015, Neuron 88:357-366) or the location of the reward (our own observations). Also, the cue-less belt that we used for the initial training was shorter than the cued belts (1.5m versus 2m) such that this possible frame of reference was also novel when the recordings were started.

3. Lines 140ff: these classification criteria are not presented here for the first time, but a recapitulation of the authors’ pervious study. I would suggest to shorten it and/or clearly refer to the original source.

As suggested by the reviewer, we have shorten the description and clearly refer to our previous study in the text. More details were put in the figure legend of Supplementary Figure 2.

4. The authors speculate that cells exhibiting multiple firing fields might correspond to young GCs. This would indeed be in line with the work from the Losonczi group (Danielson et al papers). However, given their distinctive electrophysiological properties, I doubt that mature and young GCs would end up in the same classification cluster.

We agree with the reviewer that young GCs might indeed differ from mature GCs in terms of autocorrelogram, gamma phase and gamma power. We are now more careful regarding the identity of GCs with multiple firing fields. We suggest that they might be young GCs or simply mature GCs that are more excitable. The new model implementation that includes the non-grid spatial cells in EC is compatible with both scenarios as it does not require mature-to-young GC connections.

5. Line 173: cells with low firing have not been included. How low? Could spatial maps be computed and statistical significance of spatial modulation assessed? These data could still be interesting, considering the sparseness of the DG code. Even single spikes could be meaningful (and worth reporting) here, given the strength of downstream detonator synapses.

We used a threshold of n = 50 spikes (this is now specified in the text). Some significant spatial modulation could be seen for very few of these cells (n = 16). Since we cannot be sure, we prefer not to include this group of cells to the group of putative GCs identified via our cell classification method. Such inclusion might not be accurate and is not expected to affect our conclusions.

6. Line 179: tactile cues shrink tubes, Velcro pieces or glues spines. It would be great to have pictures of these cues shown in the suppl materials. It would facilitate reproduction of the experimental procedures and experimental data by other groups (catalogue numbers could also be provided)

As suggested by the reviewer, we made a new figure (Supplementary Figure 4) showing pictures of the treadmill, the belt layouts and the different types of cues used. As it is visible on the pictures, the materials used for the cues are common items available in convenient stores and general electronic shops.

7. Figure 2c: I would suggest to present the panels in the same order they are referred to in the text (lines 183ff) or vice versa

We have modified the ordering in the text, as suggested by the reviewer.

8. Lines 206: n=4. What does it refer to? N=4 animals or sessions or animals?

Some of the n in that paragraph were corrected and the items they refer to is now specified. We thank the reviewer for pointing out this issue.

9. Line 268: ‘field extension is preceded by a gradual reduction in the in-field rate’ I do not really see it in the corresponding figure 4c. Both transitions appear equally sharp to me. Could maybe the authors quantify that?

We have added statistics in the text for the gradual decrease in in-field rate preceding the sharp place field extinctions (r = -0.75, p = 5e-10, Pearson’s correlation).

10. Line 283: ‘new set of landmarks’. Please specify, ideally with pictures (see comment 4 above)

The pictures of the new set of landmarks are included in the new Supplementary Figure 4.

11. Line 487: Hebbian synaptic plasticity and postsynaptic firing are referred to in the context of place field emergence. This could be embedded into the Bittner et al NatNeursc 2015; Diamantaki et al CurrBiol 2016; and Monaco et al., NatNeurosc 2014. Postsynaptic firing of principal cells (mediated by dishinibition?) could indeed play a crucial role here, also according to the behavioral data from the Knierim paper (i.e. potentiation of place fields during novelty-induced head-scanning behavior; Monaco et al., 2014).

We thank the reviewer for pointing out these references. We have considerably modified the discussion, and these studies, which were quite insightful, are now incorporated (paragraph 4).

12. Lines 512ff: the authors speculate that young GCs could receive inputs from mature GCs. Do the authors have evidence for monosynaptic excitatory connectivity between the putative mature GCs and the putative young GCs (as early speculated, the cells with multiple firing fields, line 303)?

We did not find any monosynaptic excitatory connections between single and unspecific GCs, using the crosscorrelogram analysis for synaptic pairs. However, it should be note that the sample size for this analysis is not very large given that the number of simultaneously recorded pair of single and unspecific GCs is not very large (n = 659 possible pairs), that mature-to-young GC connections might be rare, and that large spike counts are required for detecting synaptic pairs from crosscorrelograms (this is typically possible when an interneuron is the postsynaptic cell).

We thank the reviewer for his careful reading of the manuscript and insightful comments.

**Reviewer comments, second version:**

Reviewer #1 (Remarks to the Author):

The finding that the changes in responsiveness seem to be so slow it still somewhat unexpected, but the points made in my first review have been satisfactorily addressed.


Reviewer #2 (Remarks to the Author):


The authors have addressed most of my comments. The revised version is much improved and now more thoroughly considers previously published work. There are a few remaining points where previously raised concerns were only partially addressed.
(1) Abstract. The abstract has not been edited to reflect any of the changes in the manuscript. (a) It is not transparent that ‘period spatial intervals’ of GC cell representations are only seen in the context of periodically spaced landmarks. (b) Only the model that integrates LVCs and grid cells is mentioned while there is no reference to the more comprehensive model that includes non-grid cells.
(2) 1st paragraph of introduction mentions an emerging consensus, but includes two conflicting statements: ‘MCs … that strongly remap in response to small changes’ and ‘MCs … that largely overlap across contexts’. The contradictory statements are not consistent with the idea that a consensus has emerged.
(3) Last paragraph of introduction begins with the phrase ‘To test these hypotheses, …’ but previous paragraphs do not clearly state a hypothesis. Please state which hypotheses were tested.
(4) Discussion points should be avoided in the results section, in particular when not strongly supported by data.

‘…, reminiscent of grid cell activity patterns in linear alleys (interestingly, only a spatial period matching a third of the bell length was observed…)’. In this statement, the authors fail to take into consideration that the entorhinal grid cells in the hairpin maze showed – after resetting at the start of each turn – periodicity without the presence of additional landmarks along the alley. In contrast, the periodicity on the belt only emerged with landmarks in a periodic arrangments when cells could have responded to every second landmark. Without further tests of GC firing patterns in the absence of landmark periodicity, any direct comparison with grid cells is not appropriate in this paragraph. While more context is provided when this topic is discussed in the Discussion section, a statement needs to be added there that explicitly mentions that the periodicity of GC firing pattern has so far only been observed in conditions when there was also periodicity of landmarks, and the paragraph in the discussion starting with ‘The conversion of place field types …’ should be modified accordingly

‘Given that young adult-born cells are more excitable, …’ Again, this interpretation is speculative and should be omitted from the results section and only mentioned in the discussion section, where more context can be provided and alternative possibilities can be considered.

‘Hence, MC spatial activity was likely not generated by GC inputs (..) but rather by other afferents such as CA3, …’ The very next paragraph shows that it is likely not CA3 inputs, and the statement should thus be modified accordingly. Perhaps it would work better to move the statement until after the paragraph that describes the CA3 results.

(5) (the number of EC grid cells and LV cells was reduced to 150 …). Not clear if both cell types combined were 150 or if each cell type was 150.
(6) Discussion. ‘The involvement of a Hebbian form of synaptic potentiation is largely suggested …’ Given the recent results by the Magee group that stimulation can result in place fields by non-Hebbian mechanisms, there should be additional consideration whether the cited studies are sufficient to assert that a the plasticity mechanisms was indeed Hebbian.
(7) The final paragraph is extremely vague and does not summarize any of the main findings of the paper. It also makes the statement that ‘the possibility remains that the DG also forms non-spatial representations’, which is incorrect because non-spatial DG representations have already been described. The paragraph should be rewritten to focus on the main conclusions that can be drawn from the data in the manuscript.


Reviewer #3 (Remarks to the Author):

The authors have satisfactorily addressed my comments.

**Author rebuttal, second version:**

Reviewer #1 (Remarks to the Author):

The finding that the changes in responsiveness seem to be so slow it still somewhat unexpected, but the points made in my first review have been satisfactorily addressed.

Reviewer #2 (Remarks to the Author):

The authors have addressed most of my comments. The revised version is much improved and now more thoroughly considers previously published work. There are a few remaining points where previously raised concerns were only partially addressed.

Abstract. The abstract has not been edited to reflect any of the changes in the manuscript. (a) It is not transparent that ‘period spatial intervals’ of GC cell representations are only seen in the context of periodically spaced landmarks.

We have replaced ‘**periodic spatial intervals**’ by ‘**spatial patterns**’ to avoid the suggestion of grid-cell mechanism in the abstract

(b) Only the model that integrates LVCs and grid cells is mentioned while there is no reference to the more comprehensive model that includes non-grid cells.

We have replaced “**grid cell inputs**” by “**spatial inputs**”, such that the text now apply to the findings from all the models.

(2) 1st paragraph of introduction mentions an emerging consensus, but includes two conflicting statements: ‘MCs … that strongly remap in response to small changes’ and ‘MCs … that largely overlap across contexts’. The contradictory statements are not consistent with the idea that a consensus has emerged.

We have replaced ‘**emerging concensus**’ by ‘**emerging picture**’. In addition, we have replaced the formulation ‘**that strongly remap in response to**’ with ‘**that are strongly altered by**’, since the meaning of the term ‘remap’ was source of confusion.

(3) Last paragraph of introduction begins with the phrase ‘To test these hypotheses, …’ but previous paragraphs do not clearly state a hypothesis. Please state which hypotheses were tested.

We have modified the beginning of the sentence. The sentence now start as follow:

**“To investigate the development of DG spatial representations during the learning of an environment,…”**.

Two sentences later, the sentence that introduce the model start as follow: **“To test the contributions of competitive learning, EC object/spatial inputs and MC inputs, …”**

(4) Discussion points should be avoided in the results section, in particular when not strongly supported by data.

‘…, reminiscent of grid cell activity patterns in linear alleys (interestingly, only a spatial period matching a third of the bell length was observed…)’. In this statement, the authors fail to take into consideration that the entorhinal grid cells in the hairpin maze showed – after resetting at the start of each turn – periodicity without the presence of additional landmarks along the alley. In contrast, the periodicity on the belt only emerged with landmarks in a periodic arrangments when cells could have responded to every second landmark. Without further tests of GC firing patterns in the absence of landmark periodicity, any direct comparison with grid cells is not appropriate in this paragraph.

We followed the suggestion of the reviewer and removed that statement from the results.

While more context is provided when this topic is discussed in the Discussion section, a statement needs to be added there that explicitly mentions that the periodicity of GC firing pattern has so far only been observed in conditions when there was also periodicity of

landmarks, and the paragraph in the discussion starting with ‘The conversion of place field types …’ should be modified accordingly

We now understand the reviewer’s point regarding the potential modulation of periodic cells by triplets of every other landmark, which appear indeed periodic. We did not realize this feature. It is still valid that the periodic cells would need to use a spatial distance information, in addition to landmark input, to explain the generation of these 3 periodic fields. Such distance information could come from grid cells.

In the Discussion, we now explicitly mention the periodicity of the landmarks as a potential mechanism. The relevant section read as follow:

“Such periodic activity pattern was so far unseen in the hippocampus and may have emerged in the treadmill because of the novelty of the environment (considering that periodic cells almost completely disappeared after day 1), the oversimplification of the spatial information **and/or the fact that some of the landmarks on the belt were periodically interspaced (Note the periodic arrangement of every second landmarks). A possible scenario is that the periodic GCs originated from the combination of landmark-vector mechanisms and spatial distance information, the latter accounting for the selection of encoded landmarks based on landmark spatial periodicity rather than identity.**”

‘Given that young adult-born cells are more excitable, …’ Again, this interpretation is speculative and should be omitted from the results section and only mentioned in the discussion section, where more context can be provided and alternative possibilities can be considered.

We followed the suggestion of the reviewer and removed that statement from the results.

‘Hence, MC spatial activity was likely not generated by GC inputs (..) but rather by other afferents such as CA3, …’ The very next paragraph shows that it is likely not CA3 inputs, and the statement should thus be modified accordingly. Perhaps it would work better to move the statement until after the paragraph that describes the CA3 results.

We followed the Reviewer’s suggestion.

The ending of the first paragraph is now: “**Hence, MC spatial activity was likely not generated by GC inputs (consistent with^14^)**.”

The ending of the next paragraph is now: “**suggesting that MC modulation by the landmarks was likely not caused by CA3 input but by other inputs such as semilunar granule cells^26^ or the EC^27,29^** ”

(5) (the number of EC grid cells and LV cells was reduced to 150 …). Not clear if both cell types combined were 150 or if each cell type was 150.

We have reformulate that statement as follow: “ **the numbers of grid cells and LV cells were each reduced to 150** “

(6) Discussion. ‘The involvement of a Hebbian form of synaptic potentiation is largely suggested …’ Given the recent results by the Magee group that stimulation can result in place fields by non-Hebbian mechanisms, there should be additional consideration whether the cited studies are sufficient to assert that a the plasticity mechanisms was indeed Hebbian.

For the place field induction experiments carried in the dentate gyrus (Diamantaki et al. 2016), the distance between created field and stimulation site was < 10 cm for all the cells that successfully developed place fields (n = 10 cells). This suggest that the inputs reinforced were relatively coincident with the electrical stimulus, consistent with a Hebbian rule for synaptic potentiation.

From the recent work of the Magee group, it indeed appear that, in CA1, strong dendritic depolarization eliciting large plateau potentials can potentiate inputs that were active seconds before or after the depolarization. This form of synaptic plasticity is therefore less dependent on pre and postsynaptic coincident activity than previously reported LTP mechanisms (however, it is not necessarily non-Hebbian given that pre and postsynaptic coincident activity is still required to some degree and that the strength of potentiation increased with the degree of pre and postsynaptic coincident activity).

We have reformulate the relevant sentence as follow:

“**The involvement of a Hebbian form of synaptic potentiation is suggested by the observation that GCs receiving electrical stimulations tend to develop place fields in the stimulus locations^50^ (Note that a form of synaptic plasticity depending less on pre and postsynaptic coincident activity and more on non-linear dendritic response dynamics is believed to take place in CA1^51-53^).** ”

(7) The final paragraph is extremely vague and does not summarize any of the main findings of the paper. It also makes the statement that ‘the possibility remains that the DG also forms non-spatial representations’, which is incorrect because non-spatial DG representations have already been described. The paragraph should be rewritten to focus on the main conclusions that can be drawn from the data in the manuscript.

As the Reviewer suggested, we have rewritten the last paragraph, focusing on our findings. The last paragraph now reads as follow:

“**Hence, our findings suggest that a slow integration of spatial cell and landmark-vector cell inputs, achieved via competitive learning, is the mechanism underlying both the emergence of spatial representations and the continuous mapping of the space by GCs, while a function of MCs is to ensure a uniform distribution of GC representations. As a result, the DG may generate stable maps of the environments that embed object information and that are critical to distinguish similar (but not identical) contexts and to detect slight changes in object layouts. Further work may test whether competitive learning mechanisms operate on the whole DG network or on the level of DG subnetworks associated with distinct information, time scales and cell morphology^62^.**”

We are very thankful to the Reviewer for these constructive comments.

Reviewer #3 (Remarks to the Author):

The authors have satisfactorily addressed my comments.

**Reviewer comments, third version:**

**Author rebuttal, third version:**
